# Human Labor Pain Is Influenced by the Voltage-Gated Potassium Channel K_V_6.4 Subunit

**DOI:** 10.1016/j.celrep.2020.107941

**Published:** 2020-07-21

**Authors:** Michael C. Lee, Michael S. Nahorski, James R.F. Hockley, Van B. Lu, Gillian Ison, Luke A. Pattison, Gerard Callejo, Kaitlin Stouffer, Emily Fletcher, Christopher Brown, Ichrak Drissi, Daniel Wheeler, Patrik Ernfors, David Menon, Frank Reimann, Ewan St. John Smith, C. Geoffrey Woods

**Affiliations:** 1 University Division of Anaesthesia, University of Cambridge, Addenbrooke’s Hospital, Hills Road, Cambridge CB2 0QQ, UK; 2 Cambridge Institute for Medical Research, Wellcome Trust MRC Building, Addenbrooke’s Hospital, Hills Road, Cambridge CB2 0QQ, UK; 3 Department of Pharmacology, Tennis Court Road, Cambridge CB2 1PD, UK; 4 Wellcome Trust-MRC Institute of Metabolic Science, Addenbrooke’s Hospital, Hills Road, Cambridge CB2 0QQ, UK; 5 Department of Psychological Sciences, Institute of Psychology, Health and Society, University of Liverpool, Liverpool L69 7ZA, UK; 6 Department of Medical Biochemistry and Biophysics, Karolinska Institutet, 171 77 Stockholm, Sweden

**Keywords:** labor pain, nociception, pain, Kv6.4, quantitative sensory testing, DRG neuron, exome sequencing

## Abstract

By studying healthy women who do not request analgesia during their first delivery, we investigate genetic effects on labor pain. Such women have normal sensory and psychometric test results, except for significantly higher cuff pressure pain. We find an excess of heterozygotes carrying the rare allele of SNP rs140124801 in *KCNG4*. The rare variant K_V_6.4-Met419 has a dominant-negative effect and cannot modulate the voltage dependence of K_V_2.1 inactivation because it fails to traffic to the plasma membrane. *In vivo*, *Kcng4* (K_V_6.4) expression occurs in 40% of retrograde-labeled mouse uterine sensory neurons, all of which express K_V_2.1, and over 90% express the nociceptor genes *Trpv1* and *Scn10a*. In neurons overexpressing K_V_6.4-Met419, the voltage dependence of inactivation for K_V_2.1 is more depolarized compared with neurons overexpressing K_V_6.4. Finally, K_V_6.4-Met419-overexpressing neurons have a higher action potential threshold. We conclude that K_V_6.4 can influence human labor pain by modulating the excitability of uterine nociceptors.

## Highlights

• *KCNG4* variant highly prevalent in women requiring no analgesia in childbirth
• *KCNG4* variant encodes K_V_6.4Met-419; K_V_6.4 is a silent subunit modifying K_V_ activity
• K_V_6.4Met-419 is retained in the cytoplasm and acts in a dominant-negative manner
• K_V_6.4Met-419 overexpression results in hypoexcitable sensory neurons

## Graphical Abstract

**Figure:**
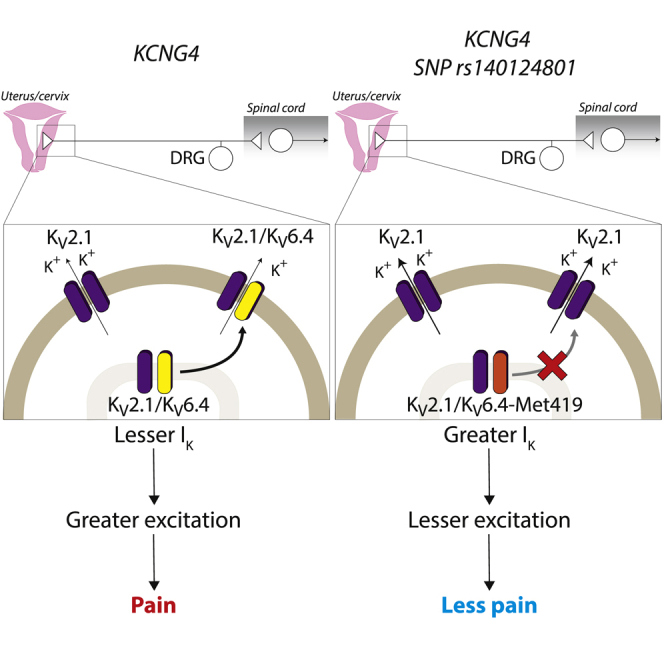

## Teaser

Lee et al. find that the K_V_6.4 variant K_V_6.4Met-419 is highly prevalent in women requiring no analgesia in childbirth and that K_V_6.4Met-419 is retained in the cytoplasm, preventing it from modifying K_V_2.1 channel activity, which results in hypoexcitable sensory neurons.

## Introduction

All eutherians (placental mammals) experience contraction of the uterus and discomfort during parturition. Although this discomfort is universal in eutherians, it appears to be most marked in humans (Maul, 2007). The severity of labor pain is considered a consequence of positive sexual selection in modern humans (with females seeking the cleverest mate), which has led to the human brain (and head) being three times the relative size of our nearest primate relatives (Sherwood et al., 2012). Despite neoteny (birth of offspring in a relatively immature state), this imposes a need to deliver a large neonatal head through the birth canal, causing labor pain (Gruss and Schmitt, 2015). Although labor pain is clearly linked to uterine contractions and cervical distension, the generation of this visceral signal and the sensory afferents involved are poorly understood (Labor and Maguire, 2008).

Although there are well-established ethnic, social, and cultural factors that influence the experience and expression of pain during labor (Whitburn et al., 2017), broader genetic effects on labor pain may also exist. For example, women with the very rare Mendelian disorder congenital insensitivity to pain due to bi-allelic non-functional mutations in *SCN9A* (MIM: 243000) do not report labor pain or require analgesics during labor (Haestier et al., 2012). *SCN9A* encodes for the voltage-gated sodium channel Na_V_1.7, expressed selectively in nociceptive and autonomic neurons, and mutations in *SCN9A* have well-documented roles in causing extremely painful or painless phenotypes (Bennett et al., 2019). The painlessness conferred by loss-of-function *SCN9A* mutations is clearly maladaptive and can be associated with severe injury during human parturition (Wheeler et al., 2014).

Our aim here was not to discover very rare Mendelian mutations that cause extreme painlessness, for example, congenital insensitivity to pain. Instead, the genetic analyses employed here are optimized for investigation of phenotypes that require an environmental trigger and genetic predisposition and that will not appear to have a Mendelian inheritance pattern unless the triggering event is frequent (Stouffer et al., 2017). This approach is suited for the study of labor pain, which may be considered nociceptive in nature, with parturition serving as a visceral stimulus. We sought to identify functional SNP alleles that are over- or under-represented in a cohort of women who did not request or use analgesics that were available and offered to them during labor: an observable behavioral phenotype considered highly unusual in hospital maternity units in the United Kingdom, particularly for spontaneous delivery of term nulliparous women. Quantitative sensory testing, performed with our study cohort, suggests a general increase in pain thresholds and tolerance compared with controls, but only the increase in the cuff pressure pain threshold survived statistical significance after adjustment for multiple comparisons. We next assessed the allele frequencies of all (genome-wide) protein-changing single nucleotide polymorphisms (SNPs) in these women compared with population frequencies. We found that the voltage-gated potassium channel (K_V_) modifier *KCNG4* (K_V_6.4) SNP rs140124801 rare allele c.1255G>A p.(Val419Met) was over-represented. Finally, we demonstrate the effects of this rare K_V_6.4-Met419 variant on sensory neuron excitability and reveal a mechanism through which uterine nociception and, hence, labor pain can be attenuated in humans.

## Results

### Identifying Women Who Did Not Require Analgesics during Labor as Nulliparous Parturients: The Test Cohort

1,029 potential subjects were identified from 8 maternity units in the United Kingdom over a 3-year period. Each potential subject was invited to contact researchers, as chronologically ascertained. 383 women responded and were screened via telephone (Figure S1A). Key inclusion criteria were healthy Caucasian women who experienced term (beyond 37-week gestation) and spontaneous vaginal delivery as nulliparous parturients without any use or request of any form of systemic or regional analgesia (spinal or epidural). We excluded women who had major diseases or co-morbidities that are known to influence labor pain or pain in general. 189 women met the full eligibility criteria (Table S1), returned written consent, and donated 10 mL of blood (collected at their local hospital) or 2 mL of saliva, sent via postal service, from which DNA was extracted.

Of the women who donated DNA, 39 consented to a subsequent study of psychometrics and quantitative sensory testing. These women comprised a subset of the genetic discovery cohort for a case-controlled study (Figure S1B). For the control cohort, we recruited 33 women who were matched in age at delivery of the firstborn and location of maternity service but who used analgesics during labor and delivery of their firstborn (Table S1). There were no significant differences in the means of newborn weight or head circumference between test and control cohorts (Table 1).

**Table 1:** Key Characteristics of the Test Cohort of Women Who Did Not Request or Require Analgesics during Nulliparous Term Spontaneous Labor and Control Subjects Who Did

Characteristics (at Delivery of First-Born)	Test Cohort			Control Cohort			p Unadjusted	p Adjusted^∗^	CI5	CI95
	n	Mean	SD	n	Mean	SD				
Age (years)	39	32.83	4.18	33	31.94	3.98	0.33	N/A	−2.73	0.93
Head circumference of newborn (cm)	26	34.00	0.98	^+^24	34.46	0.97	0.10	N/A	−0.10	1.01
Weight of newborn (g)	38	3362	434.1	33	3384	419.2	0.83	N/A	−180.90	224.76
Characteristics (at Research Visit)	Test Cohort			Control Cohort			p Unadjusted	p Adjusted^∗^	CI5	CI95
	n	Mean	SD	n	Mean	SD				
Age (years)	39	36.26	4.18	33	36.45	4.11	0.62	N/A	−1.48	2.46
Upper arm diameter at assessment (cm)	39	28.54	3.60	33	29.23	3.63	0.43	N/A	−1.03	2.41

Sensory and Pain Thresholds										

Cold detection (°C)	39	30.45	0.93	33	30.35	0.95	0.79	N/A	−0.42	0.25
Warmth detection (°C)	39	34.43	0.99	33	34.97	0.87	0.002	0.012	0.28	1.05
Cuff pressure detection (mmHg)	39	28.44	7.79	33	27.10	8.38	0.51	N/A	−5.00	1.33
Cold pain (°C)	39	11.64	8.26	33	16.88	9.03	0.02	0.114	1.17	9.73
Heat pain (°C)	39	44.08	2.85	33	42.36	3.40	0.018	0.103	−2.92	−0.27
Cuff pressure pain (mmHg)	39	166.7	54.74	33	113.03	42.96	0.00002	0.00012	−77.03	−30.13

Pain Tolerance (Cold Immersion)										

Pre-immersion hand temperature (°C)	34	30.46	1.95	33	30.82	1.66	0.42	N/A	−0.53	1.24
Post-immersion hand temperature (°C)	33^b^	17.92	4.72	33	20.51	3.54	0.02	0.12	0.40	4.60
Latency to hand withdrawal (s)	36^b^	77.03	71.82	33	44.11	55.73	0.03	0.14	−38.0	−0.0000
Peak pain occurrence (0–100 mm)	35^b^	80.19	27.39	33	79.04	28.99	0.71	N/A	−5.50	4.00
Peak pain intensity (0–100 mm)	35^b^	54.29	17.26	33	65.82	13.20	0.004	0.02	3.20	18.1
SFMPQ (sensory)	36	8.47	3.82	33	10.97	4.00	0.010	0.049	0.62	4.38
SFMPQ (affective)	36	1.00	1.53	33	1.24	1.35	0.26	N/A	−0.00002	0.99995

n, number of participants; SD, standard deviation; ^∗^, Sidak’s correction; CI5, 5% confidence interval; CI95, 95% confidence interval; SFMPQ, short-form McGill’s pain questionnaire.

^a^Missing clinical record.

b Equipment failure or unavailable.

### Cognitive and Emotional Functions Are Normal in the Test Cohort

Psychometrics, comprising validated questionnaires and computerized cognitive assessments, were employed to quantify mood, beliefs, and personality traits that can influence pain in experimental or clinical settings. The questionnaires included were the Hospital Anxiety and Depression Scale (HADS; Zigmond and Snaith, 1983), Pain Catastrophizing Scale (PCS; Sullivan et al., 1995), Multidimensional Health Locus of Control Scale (MHLC; Stevens et al., 2011), and Life Orientation Test – Revised (LOTR; Scheier et al., 1994). Computerized cognitive assessments were implemented in CANTAB (Cambridge Cognition, UK; Robbins et al., 1998). There were no significant differences in psychological or cognitive measures between the control and test cohorts (Table S2).

### Experimental Pain Thresholds and Tolerance Are Increased in the Test Cohort

Next, we quantified sensory detection and pain thresholds to cold, heat, and mechanical pressure. Thermal stimuli were delivered using a skin thermode applied to the forearm. Mechanical pressure was exerted via compression of the upper arm by a sphygmomanometer cuff. There were no significant differences in the detection thresholds of cold or cuff pressure in the test and control cohorts to suggest sensory deficits or impairments pertaining to those stimuli in the test cohort (Table 1; Figure S2A). Warmth detection thresholds were very slightly but significantly lower in the test cohort compared with controls (0.54°C difference), but all individual values fell within established norms for the general population (Rolke et al., 2006a).

The test cohort had increased pain thresholds to heat, cold, and cuff pressure at an unadjusted significance level of p < 0.05 compared with controls (Figure S2A). There was a very striking increase of over 50 mmHg in the cuff pressure pain threshold (p = 0.00002, uncorrected; p = 0.00012, Sidak’s correction; Table 1), suggesting that this characteristic might be relevant to the lack of analgesic requirement during nulliparous labor in the test cohort.

During testing for tolerance to pain from immersion of a hand in cold water (3°C), compared with controls, the test cohort showed increased hand withdrawal latency (p = 0.03, uncorrected), lower post-immersion skin temperatures (p = 0.02, uncorrected), and a lower peak intensity of pain on the 100-mm visual analog scale (VAS) (p = 0.004, uncorrected; p = 0.02, Sidak’s correction) upon later assessment (Figure S2B). The short-form McGill pain questionnaire (Melzack, 1987) revealed lower scores (p = 0.01, uncorrected; p = 0.049, corrected) for the sensory descriptors for the test group. There was no between-group difference in scores related to the affective aspects of the experimentally induced pain experienced (p = 0.26). These individual results do not survive statistical correction for multiple comparisons; further work is necessary to determine whether cold pain tolerance differs between the test and control cohorts.

### The Rare Allele of rs140124801 in *KCNG4* Is Over-represented in the Test Cohort

**Figure 1:**
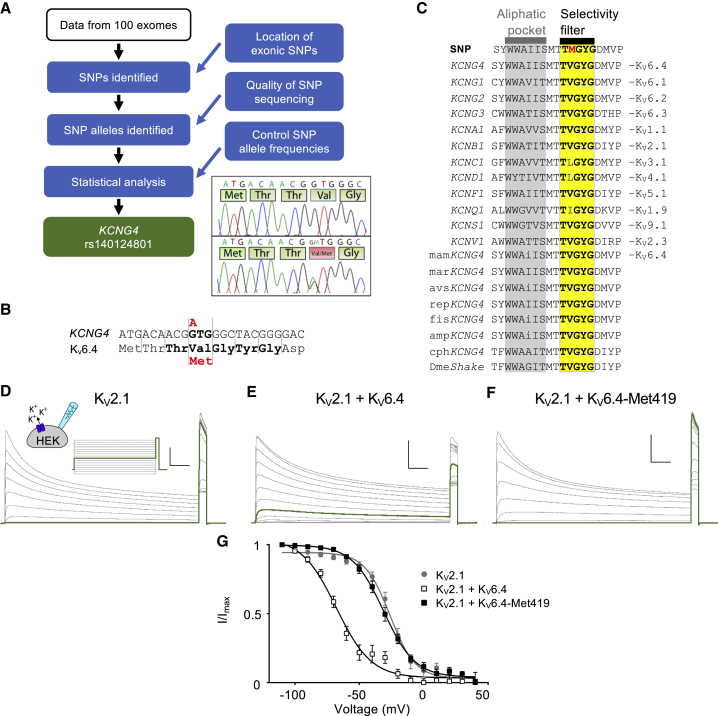
Molecular Genetics of *KCNG4* SNP rs140124801 and Analysis of K_V_2.1 Inactivation Properties (A) Summary of the genetic analysis. The resultant finding is of the SNP rs140124801 in *KCNG4*. Inset: electrophoretograms showing the alleles. (B) The nucleotide sequence of the SNP rs140124801 (NM_1.NM_172347.2), showing the altered GTG codon (boldface) and the rare allele (red). Amino acids 416–423 of K_V_6.4 (NP_758857.1) are shown below their nucleotide codons. The selectivity filter is shown in boldface, and the wild-type Val-419 is shown above Met-419. (C) Evolutionary conservation of human K_V_6.4 positions 408–426: rs140124801 alleles and representative proteins of each human K_V_ class and of K_V_6.4 in vertebrates. Invariant amino acids are capitalized. The selectivity filter TVGYG is shown in yellow and the conserved aliphatic region in gray. (D–F) Representative current recordings to determine K_V_2.1 (D), K_V_2.1/K_V_6.4 (E), and K_V_2.1/K_V_6.4-Met419 (F) steady-state inactivation properties. The applied voltage protocol is illustrated above (D). Vertical scale bar, 10 nA; horizontal scale bar, 0.5 s. Green traces indicate currents recorded during the −40 mV conditioning step. (G) Voltage dependence of steady-state inactivation of K_V_2.1 (gray filled circles, n = 9), K_V_2.1/K_V_6.4 (white squares, n = 12), and K_V_2.1/K_V_6.4-Met419 (black squares, n = 15). Symbols represent mean values, and error bars indicate SEM. Solid lines represent the Boltzmann fitted curves.

In 158 of the 189 women who did not require analgesics during their first labor, we obtained enough high-quality DNA for molecular genetic analysis (Figure S1). The chronologically first 100 such women (by date of banking DNA) constituted a discovery cohort (Figure 1A); the next 58 women constituted our replication cohort. Those in the discovery cohort had exome sequencing, from which we used the bam and bam.bai files for genome-wide SNP allele frequency assessment using the fSNPd program (Stouffer et al., 2017). The replication cohort of 58 was assessed only for SNP rs140124801 alleles using Sanger sequencing of genomic DNA.

Our discovery cohort analysis identified one ion channel SNP where the allele frequency was altered compared with the reference (Figure 1A; Table S3). The rare allele of rs140124801 in *KCNG4* was over-represented, being found in 3 instances, whereas 0.7 instances were expected (q = < 0.05, false discovery rate [FDR] corrected). We examined the individual exome results using the Integrated Genome Viewer (https://software.broadinstitute.org/software/igv/) and found that 3 individuals were heterozygous for the rare allele and confirmed this by Sanger sequencing. In the replication cohort, we found one further rare SNP rs140124801 heterozygote. For the total cohort of 158 women not requiring analgesia during their first delivery, there were 4 heterozygotes carrying the rs140124801 rare allele compared with an expected 1.1 (chi-square two-tail with Yates correction = 4.779, p = 0.0288; Figure S1A).

In case-controlled studies, we further explored whether 3 of the individuals who possess the rare *KCNG4* allele had significantly different experimental pain thresholds compared with those who did not (n = 69; Figure S1B). We investigated pain thresholds for heat, cold, and cuff pressure and found that the rare *KCNG4* allele was associated with a significantly increased cuff pressure pain threshold (p = 0.0029, uncorrected; p = 0.009, Sidak’s correction; Table S4). Although the sample size here is very small because of the rarity of the *KCNG4* allele being examined, the finding suggests that an effect of this rare allele is to increase the experimental cuff pressure pain threshold in humans. The experimental cuff pressure pain remains significantly increased in the test cohort (even with the 3 rare allele cases excluded, compared with the control group (p = 0.0029, uncorrected; p = 0.009, Sidak’s correction; Table S4) suggesting that the cuff pressure pain threshold might be relevant to labor pain. Although there are clearly other reasons for an increased cuff pressure pain threshold in individuals who do not carry the rare *KCNG4* allele, these data suggest that the rare allele of *KCNG4* may be related to the lack of analgesic requirement for the 3 subjects we identified in this study.

### The p.Val419Met Change in K_V_6.4 Impairs the Function of K_V_2.1 Heterotetramers

The rare allele of rs140124801 in *KCNG4* causes the missense change p.Val419Met encoding K_V_6.4 (from here on referred to as K_V_6.4-Met419; Figures 1A and 1B). K_V_s are tetrameric complexes, with each subunit having six transmembrane domains (S1–S6). K_V_6.4 is a member of the electrically silent group of K_V_ subunits, which cannot form functional plasma membrane-expressed homotetramers but, instead, act as modulators of K_V_2 subunits (Bocksteins and Snyders, 2012). Indeed, K_V_6.4 is known to heterotetramerize with K_V_2.1 in a 1:3 stoichiometry (Bocksteins et al., 2017). Valine 419 is in the pore-forming S5-S6 linker and is part of the highly conserved K^+^ selectivity filter consensus sequence (T**V**GYG; Figure 1C), in which the equivalent position is always occupied by a branched-chain amino acid. Although originally thought to be relatively rigid, this structure is also involved in open-pore or C-type inactivation because subtle rearrangements block the conductive path of K^+^ ions (Cuello et al., 2010). It therefore seemed likely that rs140124801 might affect K^+^ selectivity and/or inactivation; thus, we studied the electrophysiological properties of K_V_6.4-Met419 in complex with K_V_2.1 compared with the most frequent *KCNG4* allele, which possesses a valine at position 419 (K_V_6.4) in complex with K_V_2.1.

We used HEK293 cells as a heterologous expression system that does not express significant endogenous K_V_ currents (Figure S3). As expected, overexpression of K_V_6.4 or K_V_6.4-Met419 alone did not produce measurable K^+^ currents (Figure S3E). However, in cells expressing K_V_2.1 alone, outward currents were observed that were activated by potentials more positive than −40 mV and displayed slow inactivation (Figure S3A). Co-expression of K_V_2.1 with K_V_6.4 produced outward currents with similar kinetics (Figure S3D), but we observed a small shift in the voltage of half-maximal activation (V_0.5_ act) to more negative potentials. This shift was not observed when K_V_6.4-Met419 was co-expressed with K_V_2.1 (Figure S3D). The current amplitude generated was similar between wild-type K_V_6.4 or K_V_6.4-Met419 co-expressed with K_V_2.1 (Figure S3E), showing that expression of K_V_6.4-Met419 does not negatively regulate maximal current flux, over wild-type K_V_6.4, a factor that would affect sensory neuron excitability (Figure S3E). The slope factors of the Boltzmann fits did not significantly differ between the 3 groups (K_V_2.1: k = 9.5 ± 0.8, n = 13; K_V_2.1 + K_V_6.4: k = 15.9 ± 1.7, n = 14; K_V_2.1 + K_V_6.4-Met419: k = 11.0 ± 0.8, n = 13; one-way ANOVA, p > 0.05). Furthermore, the reversal potential was not significantly different between the groups (Figure S3F).

Similar to previous reports (Bocksteins et al., 2012), co-expression of K_V_6.4 resulted in a large hyperpolarizing shift in the voltage dependence of inactivation by ~30 mV compared with K_V_2.1 homomeric currents (Figures 1D, 1E, and 1G). This hyperpolarizing shift was not observed when K_V_2.1 was co-expressed with K_V_6.4-Met419 (Figures 1F and 1G). There was, however, no significant difference in the slope factor of inactivation curves between the three groups (K_V_2.1: k = 9.8 ± 1.4, n = 9; K_V_2.1 + K_V_6.4: k = 13.6 ± 2.4, n = 12; K_V_2.1 + K_V_6.4-Met419: k = 12.2 ± 1.2, n = 15; Kruskal-Wallis test, p > 0.7) or in their time courses of recovery from inactivation (Figure S3G). These data suggest loss of K_V_6.4 function as a result of the p.Val419Met mutation.

### K_V_6.4-Met419 Does Not Traffic with K_V_2.1 to the Plasma Membrane

**Figure 2:**
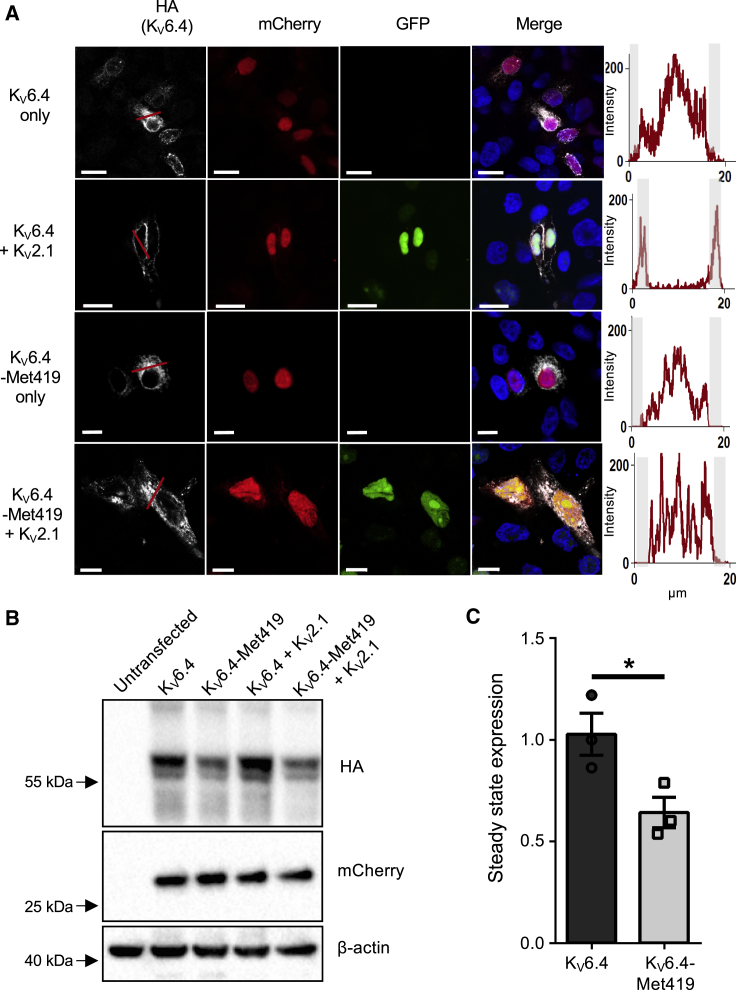
p.Val419Met Blocks K_V_6.4 from Reaching the Plasma Membrane Independent of Changes in Steady-State Expression (A) Immunofluorescence analysis of K_V_6.4 localization. In the absence of K_V_2.1, K_V_6.4 was retained in the cytoplasm (white channel, top panel) and trafficked to the cell membrane in the presence of K_V_2.1 (white channel, second row). In contrast, HA-tagged K_V_6.4-Met419 did not localize to the cell membrane in the absence or presence of K_V_2.1 expression (white channels in the third and fourth rows). Expression of K_V_2.1 is demonstrated by the presence or absence of green nuclei, expression of K_V_6.4 is displayed directly by HA tag in the white channel, and expression of the IRES vector expressing K_V_6.4 is displayed by the presence of the mCherry signal in the red channel. Graphs adjacent to each row display the intensity of the K_V_6.4 HA signal along the red line in each respective white channel; note membrane-localized peaks only in K_V_6.4 when co-expressed with K_V_2.1. Scale bars indicate 10 μm. (B) HA-tagged K_V_6.4 was transiently expressed in the presence or absence of K_V_2.1. There was a modest reduction in steady-state stability for K_V_6.4-Met419 compared with K_V_6.4. (C) Stability as assessed by densitometry of HA compared with mCherry as a control of transfection efficiency. Error bars indicate standard error. Unpaired t test (*p = 0.04).

As discussed above, K_V_6.4 forms heterotetramers with K_V_2.1 with altered biophysical properties compared with homotetrameric K_V_2.1 channels (Bocksteins, 2016; Figures 1D–1G; Figure S3). In addition, K_V_6.4 is retained in the endoplasmic reticulum in the absence of K_V_2.1, requiring expression of K_V_2.1 for trafficking to the cell membrane (Ottschytsch et al., 2005). We thus tested whether the p.Val419Met alteration might affect trafficking of K_V_6.4. For this, K_V_6.4 was cloned into a pcDNA3-based vector containing a CMV-polioIRESmCherry expression cassette and tagged with hemagglutinin (HA), and then the p.Val419Met alteration was introduced. K_V_2.1 had been cloned previously into pCAGGS-IRES2-nucEGFP, which displays a nuclear GFP signal upon transfection. To assess membrane localization, HEK293 cells were co-transfected with K_V_2.1 and K_V_6.4 and stained for HA-tagged K_V_6.4, with co-expressing cells identified by mCherry and nuclear GFP signal. K_V_6.4 was retained within the cytoplasm in the absence of K_V_2.1 expression but displayed a striking shift to the cell membrane upon co-transfection with K_V_2.1 (Figure 2A). There was no appreciable difference in the localization of K_V_6.4-Met419 in the absence of K_V_2.1, but in the presence of K_V_2.1 and in contrast to the wild-type protein, K_V_6.4-Met419 was retained intracellularly and showed no membrane localization (Figure 2A). Importantly, expression of K_V_6.4-Met419 in HEK293 cells showed only a modest reduction in steady-state stability compared with wild-type K_V_6.4, and this was not affected by co-expression with K_V_2.1 (Figures 2B and 2C).

### K_V_6.4 Is Expressed in Nociceptors that Innervate the Uterus

**Figure 3:**
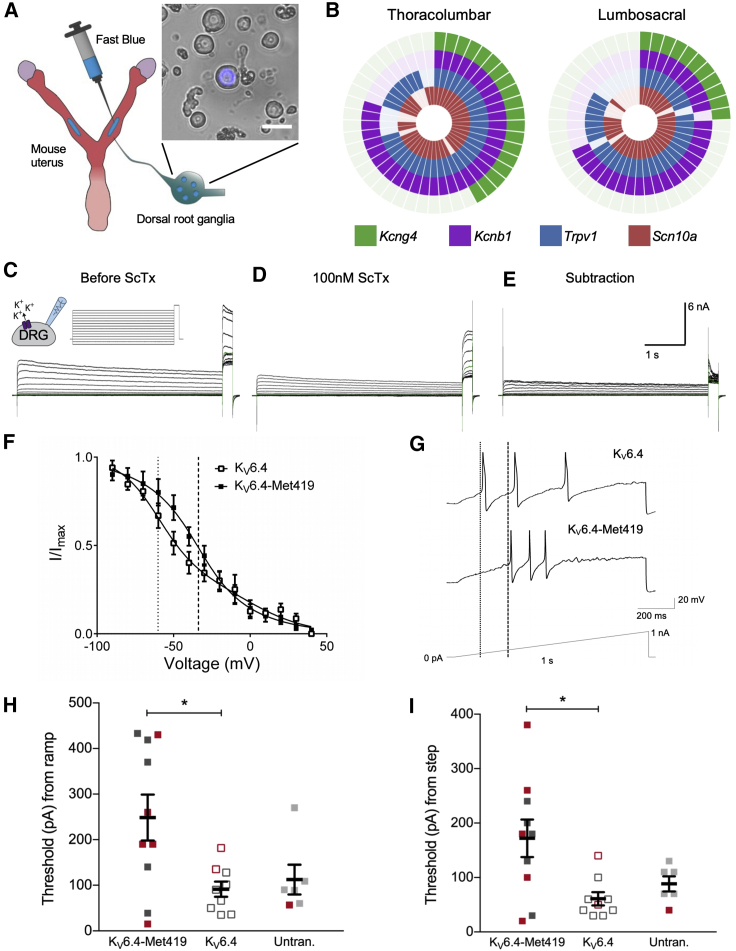
*Kcng4* Is Coexpressed with *Kcnb1* in Mouse Uterine Sensory Neurons, and Expression of K_V_6.4-Met419 in Mouse Sensory Neurons Increases the Threshold for Action Potential Discharge Compared with K_V_6.4 (A) Uterine sensory neurons were retrogradely labeled using fast blue and harvested following dissociation. Scale bar, 40 μm. (B) Co-expression analysis of TL (T12–L2, n = 44 cells) and LS (L5–S2, n = 45 cells) uterine sensory neurons expressing transcripts for *Kcng4*, *Kcnb1*, *Trpv1*, and *Scn10a*. Each segment in the wheel diagram is representative of a single cell, with a colored segment signifying positive expression. (C and D) Representative current recordings to determine the voltage dependence of steady-state inactivation of the stromatoxin-1 (ScTx)-sensitive *I*_K_ elicited by the inset voltage protocol in the absence (C) and presence (D) of 100 nM ScTx. Green traces indicate currents recorded during the −40-mV conditioning step. (E) The ScTx-sensitive *I*_K_ was obtained by subtraction of (D) from (C). (F) Inactivation curves for the ScTx-sensitive *I*_K_ for neurons transfected with K_V_6.4 (n = 8) or K_V_6.4-Met419 (n = 7). Both datasets were fit with a sum of two Boltzmann functions. The midpoints of the second components of these fits are plotted as light dashed (K_V_6.4) or heavy dashed (K_V_6.4-Met419) lines. Each point and error bars indicate mean ± SEM. (G) Representative current clamp recordings of neurons of comparable capacitance transfected with K_V_6.4 or K_V_6.4-Met419, showing action potentials evoked by ramp injection of current (0–1 nA, 1 s). The thresholds for action potential discharge are annotated with light dashed (K_V_6.4) or heavy dashed (K_V_6.4-Met419) lines. (H and I) Summary data of action potential thresholds obtained from neurons transfected with K_V_6.4 or K_V_6.4-Met419 and untransfected controls obtained via (H) a ramp protocol (0–1 nA, 1 s) or (I) a step protocol (+10 pA, 50 ms). Red points represent cells that responded to 1 μM capsaicin in voltage-clamp mode. Both recordings in (G) were from cells that were capsaicin responders. Bars indicate mean values, error bars indicate SEM, n = 6-10, ^∗^p < 0.05, one-way ANOVA with Bonferroni’s correction for multiple tests.

Altered K_V_ function produces dramatic effects upon sensory neuron excitability; K_V_7 openers (Peiris et al., 2017) and K_V_2 inhibitors (Tsantoulas et al., 2014) decrease and increase sensory neuron excitability, respectively. We hypothesized that expression of K_V_6.4-Met419 within sensory neurons innervating the uterus would alter neuronal excitability and contribute to impaired nociception. We first investigated the expression of *Kcng4* and *Kcnb1* in mouse uterine sensory neurons using single-cell qRT-PCR of sensory neurons retrogradely labeled with fast blue from the uterus (Figure 3A). Sensory innervation of the mouse uterus possesses two distinct peak densities within thoracolumbar (TL) and lumbosacral (LS) spinal segments (Herweijer et al., 2014). Therefore, fast blue-positive uterine sensory neurons were collected from dorsal root ganglia (DRG) isolated from vertebra levels T12–L2 and L5–S2. These had an average cell diameter of 31.0 ± 0.7 μm (n = 89), which is in broad agreement with studies investigating sensory neurons innervating the uterus and other visceral organs, including the distal colon (Herweijer et al., 2014; Hockley et al., 2019). Most uterine neurons expressed *Kcnb1* (TL, 82% [36 of 44]; LS, 66% [30 of 45]), and *Kcng4* mRNA was detected in a subset of uterine neurons from both spinal pathways (TL, 43% [19 of 44]; LS, 24% [11 of 45]; Figure 3B). The average cycle threshold (CT) value for *Kcng4*-expressing neurons was higher than that of *Kcnb1* (27.2 versus 16.3; Figure S4), which may indicate relatively lower expression levels. Importantly, all but one LS neuron co-expressed *Kcng4* with *Kcnb1*, suggesting that these two K_V_ subunits are predominantly present in the same uterine sensory neuron subset. We also assessed the mRNA expression of the nociceptor markers transient receptor potential vanilloid 1 (*Trpv1*) and voltage-gated sodium channel 1.8 (S*cn10a*). In *Kcng4*-positive uterine sensory neurons, *Trpv1* mRNA was present in 100% of TL and 91% of LS neurons and *Scn10a* in 95% of TL and 91% of LS neurons, suggesting that K_V_6.4 is expressed by a population of neurons capable of transducing noxious stimuli (Figure 3B).

### K_V_6.4-Met419 Causes Loss of Modulatory Function of K_V_2.1 and Decreases Neuronal Excitability in DRG Sensory Neurons

Given the high co-expression of *Kcng4* with *Kcnb1* in uterine sensory neurons, we next characterized the effect of K_V_6.4 and K_V_6.4-Met419 on sensory neuronal function. We recorded outward delayed rectifier K^+^ currents (*I*_K_) and investigated the effect of transient transfection of K_V_6.4 or K_V_6.4-Met419 on the stromatoxin-1 (ScTx)-sensitive *I*_K_; ScTx is a gating modifier of K_V_2.1, K_V_2.2, and K_V_4.2 that effectively blocks these channels (Escoubas et al., 2002) as well as K_V_2.1 heterotetramers formed with silent K_V_ subunits (Zhong et al., 2010). Through subtraction of *I*_K_ in the presence of ScTx from total *I*_K_ in the absence of ScTx, we isolated the ScTx-sensitive *I*_K_, which is predominantly dependent on K_V_2 channels (Figures 3C–3F). A diverse and heterogenous population of K_V_2 and silent K_V_ subunits is expressed in sensory neurons (Bocksteins et al., 2009; Hockley et al., 2019; Zeisel et al., 2018), and previous studies suggest that silent K_V_ subunits only heterotetramerize with K_V_2 subunits but not K_V_1, K_V_3, and K_V_4 subunits (Bocksteins, 2016). Therefore, we predicted that wild-type K_V_6.4 heterotetramerization with K_V_2.1 in sensory neurons would produce functional channels but with a hyperpolarized shift in the voltage dependence of inactivation compared with homotetrameric K_V_2.1 channels, as we (Figures 1D–1G) and others observed previously in HEK293 cells (Bocksteins, 2016). In contrast, we hypothesized that the K_V_6.4-Met419 subunit would be unable to evoke such a hyperpolarizing shift in the voltage dependence of inactivation.

By transfecting mouse sensory neurons with K_V_6.4 or K_V_6.4-Met419, we attempted to bias available K_V_2.1 into heterotetramers with K_V_6.4 subunits, increasing the probability of recording the contribution of K_V_2.1/K_V_6.4 heterotetramers to ScTx-sensitive *I*_K_. In K_V_6.4 and K_V_6.4-Met419 experiments, addition of ScTx led to a maximum reduction in outward K^+^ current at a 20-mV step potential, which did not differ significantly (K_V_6.4, 52.7% ± 3.8%; K_V_6.4-Met419, 45.1% ± 7.7%; Student’s t test, p = 0.37; Figures 3C–3E). The voltage dependence of ScTx-sensitive *I*_K_ activation was similar for neurons transfected with the K_V_6.4 or K_V_6.4-Met419 subunit (voltage of half-maximal activation [V_1/2_] = −5.4 ± 1.8 mV versus −9.8 ± 1.1 mV, and k = 8.6 ± 1.5 versus 8.9 ± 0.9, respectively; Figure S5). As observed previously (Bocksteins et al., 2009), the voltage dependence of ScTx-sensitive *I*_K_ inactivation for K_V_6.4 and K_V_6.4-Met419 experiments was multifactorial and fitted with a sum of two Boltzmann functions. In neurons transfected with K_V_6.4, the midpoint of the first component was −0.8 ± 29.5 mV, which likely correlates with homotetrameric K_V_2.1 currents. The second component possessed a midpoint of inactivation of −60.2 ± 6.6 mV (n = 8), a current that is likely a function of heterotetrameric K_V_2/silent K_V_ channels or differentially phosphorylated K_V_2 channels and in line with what others have reported for the second component of *I*_K_ in DRG neurons in the presence of ScTx (Bocksteins et al., 2009). Importantly, expression of K_V_6.4-Met419 led to a significant depolarizing shift in the second component of the voltage dependence of inactivation (−33.8 ± 2.1 mV, n = 7, unpaired t test, p = 0.003; Figure 3F), whereas the first component, attributed to homotetrameric K_V_2.1 *I*_K_, remained unchanged (−36.2 ± 3.3 mV, unpaired t test, p = 0.29; Table S5A).

We assessed the functional consequences on neuronal excitability of such a shift in the availability of K_V_2 channels toward more depolarized potentials through current clamp experiments. The threshold for action potential discharge was assessed for neurons transfected with K_V_6.4 or K_V_6.4-Met419 as well as neurons that exhibited no mCherry fluorescence from cultures exposed to either plasmid (considered untransfected). Neurons transfected with K_V_6.4-Met419 exhibited a higher threshold than those overexpressing K_V_6.4 or untransfected neurons during injection of a progressively depolarizing current (ramp protocol: 0–1 nA, 1 s); however, only the difference between K_V_6.4-Met419 and K_V_6.4 reached statistical significance (K_V_6.4, 91.6 ± 16.7 pA versus K_V_6.4-Met419, 248.6 ± 50.3 pA, ANOVA with Bonferroni multiple comparisons, p = 0.018; untransfected (Untran.), 112.5 ± 32.5 pA versus K_V_6.4-Met419, 248.6 ± 50.3 pA, p = 0.087; Figures 3G and 3H). A higher current was also required to evoke action potentials when the threshold was assessed with a step protocol (+10 pA, 50-ms injections starting at 0 pA). Similarly, only the difference between K_V_6.4 and K_V_6.4-Met419 proved to be significant (K_V_6.4, 61.1 ± 12.2 pA versus K_V_6.4-Met419, 172.0 ± 34.4 pA, ANOVA with Bonferroni multiple comparisons, p = 0.012; Untran., 88.3 ± 13.8 pA versus K_V_6.4-Met419 172.0 ± 34.4 pA, p = 0.124; Figure 3I). The ability of neurons to respond to capsaicin was also examined to identify putative nociceptors (i.e., those expressing *Trpv1*), but no obvious pattern regarding the subpopulations of nociceptive and non-nociceptive neurons within each group could be observed. Analyses of other action potential parameters revealed no further differences between neurons transfected with either K_V_6.4 construct or untransfected cells (Table S5B). Taken together, these findings demonstrate that sensory neurons expressing K_V_6.4-Met419 are less excitable than those transfected with K_V_6.4. We thus postulate that uterine primary afferent input into the pain pathway is likely to be reduced in women carrying the rare *KCNG4* SNP rs140124801 allele.

### Heterozygous K_V_6.4-Met419 Acts as a Dominant-Negative Mutation to Abolish Wild-Type Function

**Figure 4:**
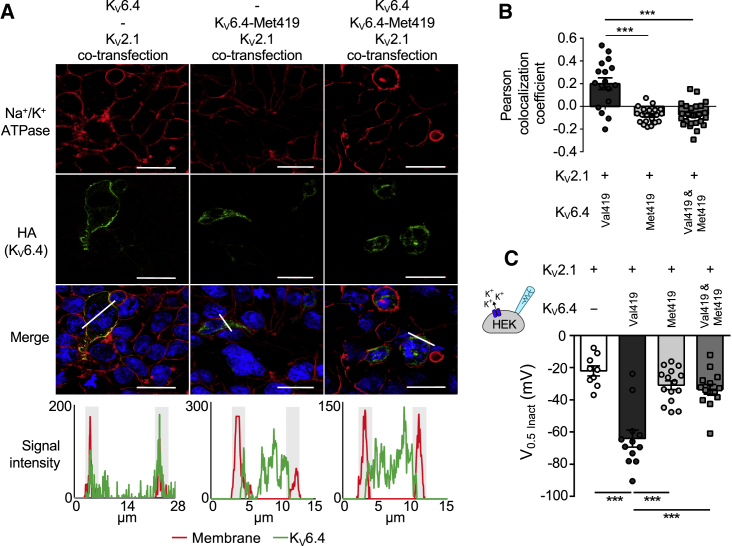
Sub-cellular Localization and Electrophysiology Analysis of the Dominant-Negative Effect of Human K_V_6.4-Met419 (A) HEK293 and HeLa cells (separate experiments) were transfected with K_V_2.1 and wild-type K_V_6.4, K_V_6.4-Met419, or equimolar concentrations of K_V_6.4/K_V_6.4-Met419. Cell membranes were stained with Na^+^/K^+^ ATPase (red channel) and HA-tagged K_V_6.4 (green channel). HA-tagged K_V_6.4 localized to the cell membrane, showing significant co-localization with Na^+^/K^+^ ATPase. K_V_6.4-Met419 and K_V_6.4/K_V_6.4-Met419 co-expression showed cytoplasmic retention of K_V_6.4 and no evidence of co-localization with Na^+^/K^+^ ATPase. The graphs display the profiles of signals for the membrane and K_V_6.4.HA along the plane of the white line in the merged image. Note that the red and green signal co-localize in the K_V_6.4 experiment and are distinct in the K_V_6.4-Met419 and heterozygote experiment. Scale bars indicate 20 μm. (B) Quantification of Pearson’s co-localization co-efficient between K_V_6.4.HA and Na^+^/K^+^ ATPase under each experimental condition. For each condition, at least 17 cells were counted from three independent experiments. (C) V_0.5_ act from inactivation protocols shown in Figures 1D–1G. Co-expression of K_V_6.4 and K_V_6.4-Met419 with K_V_2.1 failed to evoke a shift in the voltage dependence of inactivation. Bars indicate mean values, error bars indicate SEM, n = 9–15, ^∗∗∗^p < 0.001. The statistics in (B) and (C) represent one-way ANOVA with Bonferroni’s multiple comparisons test.

The SNP rs140124801 minor allele identified in healthy women not requiring analgesia during their first labor was always in a heterozygote state. We wanted to find out whether this heterozygous state has as much of an effect on K_V_2.1 as the homozygous state used in our sub-cellular localization and electrophysiology studies or whether the effect size was between homozygous K_V_6.4 and homozygous K_V_6.4-Met419. Indeed, our findings of reduced labor pain are compatible with the minor allele of rs140124801 having a dominant-negative effect or a reduced-dosage effect but incompatible when acting as a recessive. K_V_2.1 was co-transfected into HEK293 cells with equimolar concentration of K_V_6.4 and K_V_6.4-Met419 and stained for HA-K_V_6.4 and the membrane marker Na^+^/K^+^ ATPase. We found significant co-localization of K_V_6.4 with Na^+^/K^+^ ATPase at the plasma membrane but no evidence of trafficking to the cell membrane for homozygote K_V_6.4-Met419 or when K_V_6.4 and K_V_6.4-Met419 were co-transfected (Figures 4A and 4B).

Similarly, co-transfection of equimolar K_V_6.4 and K_V_6.4-Met419 with K_V_2.1 produces electrophysiological properties comparable with transfection of K_V_2.1 only; i.e., co-expression of the minor allele variant prevented the hyperpolarizing shift of the voltage dependence of inactivation produced by the major allele variant (Figure 4C).

**Figure 5:**
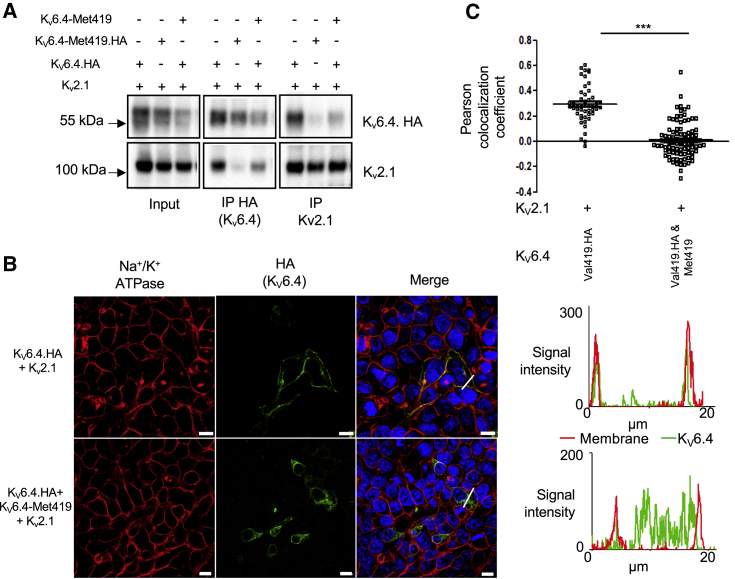
Effects of K_V_6.4-Met419 on K_V_2.1 Heterotetramerization (A) Wild-Type K_V_6.4 co-immunoprecipitates with K_V_2.1 when co-expressed in HEK293 cells (pulling down with K_V_2.1 or HA-tagged K_V_6.4). K_V_6.4-Met419 disrupts binding to K_V_2.1, and there is significantly reduced binding of HA-tagged K_V_6.4 to K_V_2.1 when co-expressed with untagged K_V_6.4-Met419. (B) K_V_6.4 traffics to the plasma membrane less efficiently when co-expressed with untagged K_V_6.4-Met419, indicating a dominant-negative effect. Scale bars indicate 10 μm. (C) Quantification of K_V_6.4 membrane localization by Pearson’s coefficient, assessing co-localization of HA and the Na^+^/K^+^ ATPase membrane marker. Data are from three independent experiments. Error bars indicate SEM, ***p < 0.001

In addition, we investigated whether K_V_6.4-Met419 might affect heterotetramerization with K_V_2.1. Co-immunoprecipitation experiments in transfected HEK293 cells demonstrate that, unlike K_V_6.4, K_V_6.4-Met419 is unable to bind to K_V_2.1 (Figures S6A and S6B). When K_V_6.4 is tagged but co-expressed with K_V_6.4-Met419 (untagged), there is notably reduced binding of K_V_6.4 to K_V_2.1 (Figure 5A). Similarly, by immunofluorescence analysis, the presence of untagged K_V_6.4-Met419 suffices to disrupt K_V_6.4 trafficking to the plasma membrane (Figures 5B and 5C).

We therefore conclude that the K_V_6.4-Met419 variant acts as a dominant-negative subunit and significantly affects the function of K_V_6.4 (and, hence, K_V_2.1) in the heterozygote state identified in our cohort of women who did not require analgesia during their first labor.

## Discussion

Parturition may be physiological and widely considered to be “natural” but remains among the most painful events in life that women can experience (Melzack, 1984). Labor pain is a complex experience with many biopsychosocial determinants, of which visceral nociception is fundamental and necessary. Although the cellular and molecular substrates for visceral nociception are ill defined in humans, ion channels that are important regulators of uterine sensory neuron excitability may determine visceral nociception and, hence, labor pain.

Labor pain is challenging, if not impossible, to model adequately in pre-clinical laboratories. Our genetic approach in humans here was not to discover very rare Mendelian mutations that cause extreme and, hence, pathological painlessness (e.g., congenital insensitivity to pain). Instead, we sought to investigate SNPs that are more common and for which frequencies in the general population are known. We hypothesized that such SNPs would be significantly over- or under-presented in a cohort of women with a less extreme but nonetheless clinically relevant phenotype. Hence, we chose to investigate healthy nulliparous women who chose and were able to manage pain from spontaneous and uncomplicated vaginal delivery of term labor without any analgesia. In this group, there were no deficits in detection of innocuous warmth, cool, or cuff grip pressure to suggest clinically relevant sensory neuropathy. There were also no differences in cognitive test battery performance, pain-relevant personality traits, or emotional function compared with controls. However, these women demonstrate increased pain and tolerance thresholds to a range of noxious stimuli and significantly so for cuff pressure pain.

Given that our painlessness phenotype is far less extreme compared with that of congenital insensitivity to pain, we did not expect that any rare SNPs discovered in this study would cause a large increase in experimental pain threshold or tolerance of all stimulus modalities. Nonetheless, there is modest evidence from a study by Carvalho et al. (2013) that a composite of these measures obtained just before induction of labor in singleton, term pregnancies predicts analgesic consumption, i.e., the volume of local anesthetic infused, in women who requested an epidural. We found that the cuff pressure pain threshold was robustly and very significantly increased in women who did not request any analgesic. Labor pain has visceral and somatic components caused by contractions of uterine viscus but also by sustained stretching or compression of the pelvic floor, perineum, and vagina (Labor and Maguire, 2008), which occur in the later stages of labor as the fetus descends and may be experienced as a continuous background pain on which rhythmic pain caused by uterine contractions is superimposed (Melzack and Schaffelberg, 1987). Although speculative, the hypothesis that women with high cuff pressure pain thresholds would report reduced intensity of continuous background pain during labor is testable.

Blinding was not feasible in our experiments, and social desirability bias may explain our overall findings of an increased threshold and tolerance of pain. However, such a bias might be expected to also significantly lower scores for self-reported pain-related traits, particularly pain catastrophizing (Sullivan et al., 1995), but that was not observed. Our data are consistent with those from other investigators who show that scores from the PCS and Fear of Pain questionnaires do not influence self-reported or behavioral measures of labor pain (Carvalho et al., 2014). Pain is a complex experience with sensory-discriminatory and affective-motivational aspects (Loeser and Treede, 2008). We found that the test cohort had lower short-form McGill’s pain questionnaire (SFMPQ) scores that pertained to the sensory but not the affective qualities of the pain experienced during cold tolerance testing. In sum, we found an increased threshold to pain from noxious stimuli (significantly so for cuff pressure) but no differences in cognitive function, personality traits, and emotional function in women who did not require analgesics during term nulliparous labor. These findings suggest that nociceptive function is altered in these women and validate their selection to discover predisposing genetic changes in sensory neurons (nociceptors) that might influence labor pain in women, a phenotype that otherwise would confidently have been expected to be highly heterogeneous.

We detected a single SNP, rs140124801, in the gene *KCNG4*, where the rare allele had a significant over-representation compared with the general population in a cohort of 158 women who had no analgesic requirement during nulliparous labor, noting that, ideally, control allele frequencies would have been generated from a matched cohort of women who did require analgesia. There were 4 heterozygotes who possess the rare allele, and data on quantitative sensory and pain testing were available for 3 heterozygotes. We found that women who possess the rare allele showed a significantly increased cuff pressure pain threshold compared with controls (Table S4). The rare allele of SNP rs140124801 causes a missense change, p.Val419Met, in K_V_6.4, a silent K_V_ subunit that forms heterotetramers with K_V_2 channels and modulates their function (Bocksteins et al., 2012). We and others show that K_V_6.4 traffics to the plasma membrane only when co-expressed with K_V_2.1 (Ottschytsch et al., 2005). In contrast, we found that the rare allele product K_V_6.4-Met419 failed to traffic to the plasma membrane when co-expressed with K_V_2.1. Moreover, K_V_6.4-Met419 failed to induce the hyperpolarizing shift in the voltage dependence of K_V_2.1 inactivation that is observed with K_V_6.4, likely indicating that the observed currents would be conducted by K_V_2.1 homotetrameric channels.

We have found that K_V_6.4-Met419 was unable to heterotetramerize with K_V_2.1. A possible explanation for this is gained from X-ray crystallography of the K_V_2.1 homotetramer (PDB: 3LNM). Each of the four K_V_2.1 monomers contributes equally to the K^+^ ion selectivity region, which is formed by the peptide backbone carbonyl groups of the amino acids TVGYG. The side chains of valine and tyrosine from each of the four monomers fits within an aliphatic pocket of the adjacent monomer (composed of amino acids WWAIIS; Figure 1C). The rare allele of SNP, rs140124801, results in valine being changed to methionine, whose side chain is too large to be accommodated by this aliphatic pocket. This may be sufficient to stop K_V_6.4 forming a heterotetramer with K_V_2.1 and would be predicted to disrupt the close packing of the peptide backbone carbonyl groups of the ion selectivity region.

For K_V_6.4 to modulate labor pain, it needs to be expressed in an appropriate part of the sensory nervous system. We focused on uterine sensory neurons, but this does not negate the possibility that K_V_6.4 also exerts influence elsewhere in the nervous system, given that *KCNG4* mRNA is also expressed in regions of the spinal cord and brain (Figure S6C). We observed K_V_6.4 expression in Trpv1- and Na_V_1.8-positive mouse uterine sensory neurons, consistent with the observation that sensory neurons innervating deep tissues display comparatively high Trpv1 expression (Malin et al., 2011). Results from unbiased single-cell RNA sequencing of mouse DRG neurons obtained from cervical to lumbar levels reveal no specific coexpression of K_V_6.4 in nociceptive Trpv1/Scn10a-expressing neurons (Zeisel et al., 2018). However, single-cell RNA sequencing of colonic sensory neurons identified that K_V_6.4 does co-localize with Trpv1 and Na_V_1.8 (Hockley et al., 2019), consistent with our findings here that K_V_6.4, Trpv1, and Na_V_1.8 are coexpressed in uterine sensory neurons from T12–L2 and L5–S2 DRG. Taken together, these data suggest that K_V_6.4 might be a marker for sensory neurons that innervate the viscera. Because of the restricted expression of *Kcng4* in a particular sensory neuron type, expression of K_V_6.4-Met419 is expected to reduce excitability specifically for this class of sensory neurons.

**Figure 6:**
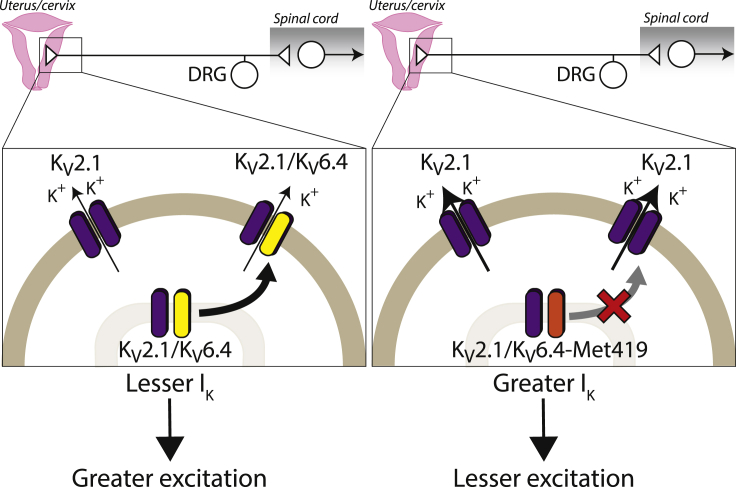
Schematic of the Mechanism by which the Rare Allele SNP rs140124801 p.Val419Met in *KCNG4* (Encoding the K_V_ Subunit K_V_6.4) Regulates Neuronal Excitability In most individuals (left panel), visceral nociceptors capable of transducing labor pain possess a combination of homomeric K_V_2.1 channels and heteromeric K_V_2.1/K_V_6.4 channels, whereas in individuals with the rare allele SNP rs140124801 p.Val419Met in *KCNG4* (right panel), K_V_2.1/K_V_6.4-Met419 heteromers fail to traffic from the cytoplasm to the plasma membrane, resulting in a greater proportion of K_V_2.1 homomeric channels. Because of their steady-state inactivation properties, K_V_2.1/K_V_6.4 heteromers have reduced availability at more depolarized membrane potentials compared with K_V_2.1 homomers, and, thus, in nociceptors expressing K_V_6.4-Met419, there is greater K_V_2.1 homomer-mediated current at depolarized membrane potentials, which reduces neuronal excitability.

For the rare allele rs140124801 to modulate labor pain, it needs to cause a significant change in K_V_6.4-influenced neuronal activity and do so in the heterozygote state. Our electrophysiology and cell trafficking studies showed that the mutant K_V_6.4-Met419, as opposed to K_V_6.4, had no effect on K_V_2.1 function, nor was it trafficked to the plasma membrane. Transfection of K_V_6.4 into mouse sensory neurons produced a more hyperpolarized voltage dependence of inactivation for the predicted heterotetrameric K_V_2/silent K_V_ channel component of *I*_K_ than when K_V_6.4-Met419 was transfected, further supporting the hypothesis that the loss-of-function K_V_6.4-Met419 results in more K_V_2.1 activity at positive voltages. K_V_2.1 is known to contribute to after-hyperpolarization duration and intra-action potential refractory period and, thus, regulate neuronal excitability (Tsantoulas et al., 2014). Hence, we anticipated that a K_V_6.4-Met419-induced deficit in K_V_2.1 function would likely result in fewer action potentials and, thus, less pain during periods of sustained nociceptor activity, such as that occurring with uterine contractions during labor. Although we did not observe a difference in after-hyperpolarization duration or action potential frequency between sensory neurons transfected with K_V_6.4 or K_V_6.4-Met419 (possibly because of the continual current injection used), we did find that a larger amount of current was required to cause K_V_6.4-Met419-expressing neurons to fire actions potentials and conclude that the mutation confers reduced neuronal excitability (Figure 6). Critically, we observed that K_V_6.4-Met419 has a dominant-negative effect on K_V_6.4 regarding modulation of the voltage dependence of inactivation for K_V_2.1. This result likely explains the reduction in labor pain seen in individuals in our cohort who were heterozygotes for the SNP rs140124801 rare allele. Although the results contained herein demonstrate the effect of K_V_6.4-Met419 on neuronal excitability, another way to demonstrate this would be to generate transgenic mice or use adeno-associated viruses to transduce sensory neurons innervating a specific target, as conducted recently with the knee (Chakrabarti et al., 2020). Using mice overexpressing K_V_6.4-Met419, we hypothesize that, like humans expressing the SNP rs140124801 rare allele, these K_V_6.4-Met419 mice might have a raised threshold to acute noxious stimuli compared with wild-type mice and potentially have a reduced chronic pain phenotype, results that would align with the known roles of K_V_ channels in mouse pain behavior; for example, knockout of K_V_9.1 leads to increased basal mechanical pain and exacerbates neuropathic pain (Tsantoulas et al., 2018).

Moreover, the importance of K_V_s in regulating neuronal excitability is highlighted by a study of induced pluripotent stem cell-derived sensory neurons (iPSC-SNs) derived from a mother and son with inherited erythromelalgia (IEM) (Mis et al., 2019). Both individuals carry the same Na_V_1.7 variant that is associated with IEM, but the frequency and duration of pain attacks differed, thus implicating further genetic variants. Whole-exome sequencing of both individuals identified a *KCNQ2* variant that encodes K_V_7.2 in the individual experiencing less pain. Interestingly, the “less pain” variant resulted in a hyperpolarizing shift in V_1/2_ for activation of the K_V_7.2-mediated M current (a major determinant of resting membrane potential [RMP]) and a more hyperpolarized RMP, making it more difficult for action potentials to be evoked in iPSC-SNs. Thus, this study, alongside ours, demonstrates the importance of K_V_ function in modulating neuronal excitability and pain experience.

There is a growing understanding of the distinctions between the neural pathways for pain from visceral and somatic tissues; each has evolved nociceptors that sense damage in different physical environments (Bertucci and Arendt, 2013). Our findings suggest a key role of K_V_6.4 in specifically regulating nociceptor excitability and, hence, pain in normal labor. K_V_6.4 is also expressed in other parts of the nervous system (Figure S6C), and its expression in non-neural tissues is unknown. However, we found that women carrying the rare allele *KCNG4* managed nulliparous labor without analgesics and had higher experimental pain thresholds but were otherwise healthy without any psychological or cognitive abnormalities. Their phenotype suggests that the loss of modulatory effects of K_V_6.4 is non-pathogenic in other parts of the nervous system and non-neural tissues. If druggable, then K_V_6.4 would be a potential target for modulating labor pain without the maternal and neonatal side effects inherent in other analgesic interventions in this setting. Our data also raise the question of whether K_V_6.4 has roles in other painful visceral disorders within and outside the female genital tract. One closely related context would be primary dysmenorrhea, which is characterized by severe pain associated with uterine contraction during menstruation (Ju et al., 2014). Further development of selective K_V_6.4 pharmacological agents is required to fully probe the role of K_V_6.4 in visceral pain.

## STAR★Methods

### Key Resources Table

**Table:** 

REAGENT or RESOURCE	SOURCE	IDENTIFIER
**Antibodies**		

Anti-HA mouse monoclonal	Biolegend	Cat# 12B12, #MMS-101P; RRID: AB_2801249
Anti-K_V_2.1 rabbit polyclonal	Alomone	Cat# APC-012; RRID: AB_2040162
Anit-K_V_2.1 mouse monoclonal	Abcam	Cat# ab192761; RRID: AB_2750677
Anti-HA rabbit monoclonal	Cell Signaling	Cat# C29F4 #3724: RRID: AB_10693385
Anti-Na^+^/K^+^ ATPase rabbit monoclonal	Abcam	Cat# Ab76020; RRID: AB_1310695
Anti β-actin mouse monoclonal	Sigma Aldrich	Cat # A2228; RRID: AB_476697
Anti-mCherry rat monoclonal	ThermoFisher	Cat # M11217; RRID: AB_2536611

**Chemicals, Peptides, and Recombinant Proteins**		

Trypsin	Sigma-Aldrich	T4799
Collagenase	Sigma-Aldrich	C5138
Bovine serum albumin	Sigma-Aldrich	B2064
Fast Blue	Polysciences	17740
Trypsin from Bovine Pancreas	Sigma-Aldrich	T9935
Collagenase, Type 1A	Sigma-Aldrich	C9891
Capsaicin	Sigma-Aldrich	M2028
Stromatoxin-1	Alomone	STS-350
FuGENE HD transfection reagent	Promega	E2311

**Critical Commercial Assays**		

CellsDirect One-Step qRT-PCR kit	Invitrogen	11753100
Amaxa Mouse Neuron Nucleofactor kit	Lonza	VPG-1001
Dynabeads Co-Immuniprecipitation Kit	ThermoFisher	14321D
*Kcng4* Taqman primer-probe	Applied Biosciences	Mm01240890_m1
*Kcnb1 Taqman primer-probe*	Applied Biosciences	Mm00492791_m1
*Trpv1* Taqman primer-probe	Applied Biosciences	Mm01246300_m1
*Scn10a* Taqman primer-probe	Applied Biosciences	Mm00501467_m1
*Gapdh* Taqman primer-probe	Applied Biosciences	Mm99999915_g1

**Experimental Models: Cell Lines**		

HeLa	Sigma Aldrich	93021013
HEK293	Sigma Aldrich	85120602

**Experimental Models: Organisms/Strains**		

C57BL/6J mice	Envigo	Wild-type

**Recombinant DNA**		

*KCNG4* cDNA clone in polioIRESmCherry w/wo HA tag	This manuscript	N/A
K_v_2.1 in pCAGGS-IRES2-nucEGFP	Gift from Prof Saitsu	Saitsu et al., 2015
*KCNG4.Met419* cDNA clone in polioIRESmCherry w/wo HA tag	This manuscript	N/A

**Software and Algorithms**		

R Studio Version 1.2.5036 for Mac	R	https://rstudio.com/products/rstudio/
pClamp (v10.3)	Molecular Devices	pClamp (v10.3)
Prism (v8)	GraphPad	Prism (v8)
Patchmaster	HEKA	heka.com
Fitmaster v2x90.4	HEKA	heka.com
Igor pro v6.37	WaveMetrics	wavemetrics.com
Patcher’s Power Tools	Max-Planck-Institut	https://www3.mpibpc.mpg.de/groups/neher/index.php?page=aboutppt
Step One version 2.3	Applied Biosystems	N/A

**Other**		

Poly-D-lysine and laminin coated coverslips	BD Biosciences	354087
Nucleofector IIb	Lonza	AAB-1001
Multiclamp 700A amplifier	Molecular Devices	N/A
Digidata 1440A	Molecular Devices	N/A
Rapid change perfusion system	Intracel	EVH-9
EPC-10 amplifier	HEKA	N/A
Pipette puller	Sutter Instruments	P-97
Lebovitz L015 Glutamax	Thermo Fisher Scientific	31415029
Poly-D-lysine coated coverslips	BD Biosciences	354086
SUPERase-inhibitor	Ambion	AM2696

### Resource Availability

#### Lead Contact

Further information and requests for resources and reagents should be directed to and will be fulfilled by the Lead Contact, Ewan St John Smith (mailto:es336@cam.ac.uk).

#### Materials Availability

Plasmid constructs generated in this study will be made available upon request, subject to ethical restrictions and Material Transfer Agreements.

#### Data and Code Availability

Clinical datasets supporting Tables 1, S2, and S3 and Figure S2 are available upon request, subject to ethical restrictions.

Fully anonymized SNP data supporting the exome analyses (Figure 1A) are provided in Table S3

### Experimental Model and Subject Details

#### Human case ascertainment and recruitment

Labour pain is a complex experience and difficult to quantify (Bergh et al., 2015). Epidurals and inhalational analgesia are currently the most effective forms of pain relief in labor (Jones et al., 2012). Hence the rate of epidural use is a recognized surrogate measure for pain in clinical trials that assess the effectiveness of other forms of analgesia in labor (Levett et al., 2016). The use of inhalational analgesia is far commoner, particularly in nulliparous parturients where labor is considered more painful. A UK survey suggests that Entonox® use in labor at 80% and first-time mothers were more likely to use labor analgesia (National Perinatal Epidemiology Unit, 2014). Hence, the phenotype for less painful labor was defined operationally as nulliparous parturients who did not request nor use epidural, inhalational or opioid-based analgesia. This behavioral definition would have captured individuals with *SCN9A* channelopathy who reported entirely painless labor (Haestier et al., 2012).

The studies commenced in October 2012 after National Research Ethics Service and Human Research Authority approval (Reference: 12-EE-0369) was granted. For the first study (Study A), potential participants were identified based at maternity units in the United Kingdom and invited via post to contact the research team. The post included an information sheet stating that the study sought “to use genetic analysis to look for variations in genes in women who do not feel as much pain as might be expected during childbirth, and determine whether such variation in pain experience might be related to genetic differences’ and the invitation was for women that ‘have had a baby and according to our records, you required minimal or no pain relief during the birth of your first child.”

All potential participants who contacted the research team were further screened via telephone interview for eligibility (Table S1). Eligible participants were posted study information and a saliva sampling kit (Oragene®-DNA, OG-500, Genotek), with a self-addressed return envelope. Participants did not receive any financial incentive for the genetic study.

In the second study (Study B), women who had consented to the genetic study and for whom exome sequencing was successful were invited to the Cambridge NIHR Clinical Investigation Ward for further study. Those who were eligible (Table S1) and consented to participate comprised the test cohort.

Women who met the study criteria but who required analgesia during labor served as case controls. Controls were informed via participant information leaflet we have *“identified women who did not use painkillers during the birth of their first child. However, we are still unsure whether they are actually less sensitive to pain. In order to find out, we need to test their pain sensitivity and compare their results to women who did used an Epidural or Entonox (gas and air) for pain relief during their first labour”*. Controls were selected to match age at delivery of first-born, location of maternity unit and age at study visit. A total of 1029 invitations were sent by post. Where available, data on birth weight of baby and head circumference were recorded. The age range for the test and control cohorts were 27 to 48 years, and 28 to 44 years respectively (Table 1).

Participants were reimbursed up to maximum of 25GDP for time in addition to travel expenses for the two-hour visit. All participants and the researchers who communicated directly with them remained blind to genotype during the study.

#### Cell lines and culture conditions

HEK293 and HeLa cells were cultured in 90% Dulbecco’s modified Eagle medium (DMEM) supplemented with 10% fetal bovine serum (FBS), 100 U/ml penicillin-streptomycin (pen-strep), and 2 mM L-glutamine at 37°C, 5% CO_2_, 100% humidity. Transfections were carried out using FugeneHD transfection reagent (Promega) according to the manufacturer’s protocols. For co-expression studies, cloned K_V_2.1 and K_V_6.4 constructs were transfected at a ratio of 1:2. Cells for experiments were plated out on glass coverslips for immunostaining or 35 mm plastic dishes for electrophysiological recordings, 1-2 days prior to the experiment.

#### Animals

Adult C57BL/6J mice (Envigo), male and female, aged between 8 to 12 weeks, were conventionally housed in groups of 4-5 with nesting material and a red plastic shelter and various enrichment toys; the holding room was temperature controlled (21°C) and mice were on a 12-hour/light dark cycle with food and water available *ad libitum.* Work was regulated under the Animals (Scientific Procedures) Act 1986 Amendment Regulations 2012 following ethical review by the University of Cambridge Animal Welfare and Ethical Review Body.

### Method Details

#### Clinical questionnaires, cognitive and sensory testing

A single research assistant in the same temperature-controlled room conducted all assessments. Participants were seated for the assessment and rest breaks were provided between assessments to minimize fatigue. Instructions for each assessment were read from a written script. These assessments were completed in the following sequence: (1) questionnaires administered on paper Hospital Anxiety and Depression Scale (HADS) (Zigmond and Snaith, 1983), Pain Catastrophizing Scale (PCS) (Sullivan et al., 1995), Multidimensional Health Locus of Control Scale (MHLC) (Stevens et al., 2011) and Life Orientation Test-Revised (LOTR) (Scheier et al., 1994), (2) quantitative sensory testing (QST) to determine stimulus detection, pain and tolerance thresholds, and (3) computerized cognitive assessments implemented on CANTAB® (Cambridge Cognition, UK) (Robbins et al., 1998). Hospital Anxiety and Depression Scale (HADS) (Zigmond and Snaith, 1983), Pain Catastrophizing Scale (PCS) (Sullivan et al., 1995), Multidimensional Health Locus of Control Scale (MHLC) (Stevens et al., 2011) and Life Orientation Test-Revised (LOTR) (Scheier et al., 1994).

#### Cambridge Neuropsychological Test Automated Battery (CANTAB)

The cognitive assessments were drawn from the Cambridge Neuropsychological Test Automated Battery (CANTAB) (https://www.cambridgecognition.com/). The computerised tests required finger-tap responses via touchscreen and are largely independent of verbal instruction. CANTAB software was deployed on an XGA-touch panel 12-inch monitor (Paceblade Slim-book P120; PaceBlade Technology). The sequence of tasks employed in the study was as follows: Motor Screening Task (MOT), Spatial Working Memory (SWM), Rapid Visual Information Processing (RVIP), Intra- Extra-Dimensional Set Shift (IED) and One-Touch Stockings of Cambridge (OTS). Descriptions of each task are provided below. All tasks were performed using the index finger of the dominant hand.

#### Motor Screening Task (MOT)

Colored crosses are presented in different locations on the screen, one at a time. The participant must select the cross on the screen as quickly and accurately as possible. Outcome measures are (a) mean latency and (b) mean error, which reflect accuracy.

#### Spatial Working Memory (SWM)

The task assesses ability to retain spatial information and manipulate items in working memory. It is considered a sensitive measure of frontal lobe and executive dysfunction. This is a self-ordered task which also assesses heuristic strategy. Several colored squares (box) are displayed in random locations on the touch screen. There is pre-set number of boxes with a blue token. The participant taps on a box to uncover a blue ‘token’ and place that token into a ‘bin’. The participant must remember which box has been tapped or emptied. The number of boxes is gradually increased until the participants is searching for tokens in a total of eight boxes. The color and position of boxes used are changed from trial to trial to discourage use of stereotyped search strategies. Outcome measures are (a) strategy, for which the fully efficient strategy would result in no boxes being revisited. A high score represents poor use of this strategy and a low score equates to effective use, and (b) total errors, which is the number of times a box is selected that cannot contain a blue token and therefore should not have been visited by the subject.

#### Rapid Visual Information Processing (RVP)

A white box is shown in the center of the screen, inside which digits from 2 to 9 appear in a pseudo-random order, at the rate of 100 digits per minute. Participants are asked to detect target sequences of digits (for example, 2-4-6, 3-5-7, 4-6-8) and respond by tapping on a button-box as quickly as possible. Outcome measures are (a) sensitivity index A’, which reflects how good the subject is at detecting target sequences, regardless of error tendency A score close to +1.00 indicates that a high true positive rate, and (b) response criterion B’, which reflects the tendency to respond regardless of whether the target sequence is present. A score close to +1.00 indicates a high true negative rate.

#### Intra-Extra Dimensional Set

This task assesses visual discrimination and attentional set formation maintenance, shifting and flexibility of attention. IED task requires participants to learn the rule and select the correct icon (a specific shape or line). The task builds in complexity as distractors are added and the rule changes. The rule changes are both intra-dimensional (e.g., shapes are still the relevant set, but a different shape is now correct) and extra-dimensional (e.g., shapes are no longer the relevant set, instead one of the line stimuli is now correct). Outcome measures are (a) total errors (adjusted), which is a measure of the participant’s efficiency. While she may pass all nine stages, a substantial number of errors may be made in doing so. The errors are adjusted to account for those who fail at any stage of the test and hence have had less opportunity to make errors, (b) number of stages completed, and (c) total trials (adjusted), which is the number of trials completed on all attempted stages for each stage not attempted due to failure at an earlier stage.

#### One Touch Stockings of Cambridge

This task is a test of executive function, based upon the Tower of Hanoi. The participant is shown two displays containing three colored balls. The displays are presented in such a way that they can be easily perceived as stacks of colored balls held in stockings suspended from a beam. The participant is shown how to move the balls in the lower display to copy the pattern in the upper display and completes one demonstration problem, where the solution requires one move. The participant must then complete three further problems, one each requiring two moves, three moves and four moves. Next the participant is shown further problems and must work out mentally the number of moves the solutions require and then select the appropriate box at the bottom of the screen to indicate their response. Outcome measures are (a) mean choice to correct, which is the mean number of attempts to the correct response, and (b) mean latency to correct, which is the overall latency (time required) to the correct response.

#### Quantitative sensory testing

Stimulus detection and pain thresholds for heat and cold were determined by applying a 3x3 cm^2^ thermode on the volar surface of the non-dominant mid-forearm (TSA, Medoc, Israel). The procedure was adapted from a clinical research protocol (Rolke et al., 2006b), for which the research assistant received formal training (Universitsmedizin Mannheim). Stimulus detection and pain thresholds were determined using increasing or decreasing temperature ramp of 1°C.s^-1^ from a baseline temperature of 32°C, with low and high safety cut-offs at 0 and 50°C respectively. Participants were instructed to click on a button when they first experience the required sensations. Four trials each with an inter-trial interval of 10 s were employed to assess heat and cold stimulus detection thresholds. Three trials with a longer inter-trial interval of 30 s were employed for heat and cold pain thresholds to minimize risks of burn.

Pressure detection and pain threshold were determined by cuff algometry (Vargas et al., 2006) applied to the dominant upper arm. The circumference of the upper arm was measured to determine the appropriate sphygmomanometer cuff size. A digital metronome (Korg MA-1, UK) was used to guide manual inflation of the cuff at 10 mmHg every 5 s. The participant was instructed to verbally report when the point the cuff was felt to be ‘just gripping’ and when the gripping became just about painful, at which point the cuff pressure was rapidly released. The pressures at thresholds were recorded. The participant was then asked to indicate when all evoked sensation in the arm had resolved. The entire procedure was repeated thrice.

Pain tolerance was measured as latency to withdrawal from immersion of hand in a cold water bath (3°C) (Mitchell et al., 2004). The participant was instructed to immerse her non-dominant hand and wrist into a circulating cold-water bath (RW2025G, Medline Scientific UK) and to withdraw the hand *ad libitum* when pain became intolerable. The maximum duration of cold-water immersion allowed was 180 s, after which the participant was told to remove her hand from the water bath. All participants were told that there was a maximum allowable duration for immersion for safety but not the exact duration to avoid anticipatory effects.

The skin temperature of the hand dorsum was measured (NC 150, Microlife, Switerland) within 60 s pre-immersion and 10 s post-immersion (after the hand was wiped dry). Participants were then asked to rate peak intensity of pain during immersion using a 100mm visual analog scale (VAS) with the left and right anchors labeled as ‘no pain’ and ‘worst imaginable pain’ respectively. They were also asked to estimate when the intensity of pain peaked during the period of immersion (100mm VAS; 0mm and 100mm represented the times of hand immersion and hand withdrawal respectively). Finally, participants completed the Short-Form McGill Pain Questionnaire (SQ-MPQ). The questionnaire comprises 15 pain descriptors: 11 pertain to sensory-discriminatory aspects (e.g., ‘hot-burning’), and the rest pertained to affective-motivational (e.g., ‘cruel-punishing’) of the pain experienced during cold-water immersion of the hand (Melzack, 1987).

#### Genetic analysis of non-synonymous functional single nucleotide polymorphism alleles

For the genetic analysis of the discovery cohort we used the fSNPd approach (Stouffer et al., 2017). In brief, the hypothesis is that some individuals with a defined phenotype (in our case reduced labor pain inferred by the absence of analgesia requirement during labor) could have genetic predisposition(s) that explain their difference in phenotype. To be detected, such a genetic predisposition would have to be dominantly inherited and often penetrant: this is the case with many known autosomal dominant Mendelian genetic disorders such as Tuberose Sclerosis and Neurofibromatosis type 1 where the phenotype is variable (and can be incomplete) despite an individual carrying the known pathogenic familial mutation. The fSNPd approach further hypothesizes that the phenotype will not be caused by very rare genetic mutations, but by the rare alleles of known SNPs where the allele difference is protein changing. Examples of such SNPs exist that only cause a human phenotype when the heterozygous individual is exposed to a specific environmental insult or trigger, e.g., aminoglycoside induced deafness (Prezant et al., 1993) and SNPs rs267606617 and rs267606618; and carbamazepine associated toxic epidermal necrolysis and rs3909184 (Chung et al., 2004).

An exome analysis was performed on the genomic DNA of the 100 individuals of the discovery cohort by Beijing Genomics Institute using the Agilent 51M kit sequenced to an average of 50-fold coverage, as previously described (Nahorski et al., 2018). Such an analysis does not include all coding exons of all human genes, and for this reason SNPs in some genes are not assessed, and other SNPs were not assessed in all individuals in the discovery cohort (Stouffer et al., 2017). The exome v*cf.*, bam and bam.bai files were iteratively analyzed extracting data on all SNP in or near to exons, including the depth and quality of the sequence data, and the alleles detected (Stouffer et al., 2017). For each SNP the allele frequencies were compared to normal values, and deviations assessed for significance using a Chi-square test with two tails and Yates correction. The resulting *P value*s were subject separately to a Bonferroni correction and false discovery rate (FDR) correction, as approximately 100,000 SNPs were assessed in our fSNPd method. We then filtered only for clear-cut protein changing SNPs (missense mutations predicted deleterious by SIFT, nonsense mutations, splice site mutations, start codon mutations, and within-exon deletions and duplications), as such changes are potentially more amenable to function tests of pathogenicity; reducing from 18,106 SNPs prior to SIFT and pathogenicity analysis to 3,596 afterward. We then further filtered only for SNPs within ion channel genes, as members of this group of genes have already been implicated in Mendelian pain disorders, and testing techniques for ion channel function are well established; resulting in 28 SNPs (Stevens and Stephens, 2018). For all SNPs, especially those whose rare allele frequency is < 5%, geographical and ethnic differences must be considered; rs140124801 has a rare allele frequency in EVS of 0.0051 (cohort size 6500), in gNOMAD Europeans = 0.0072 (cohort size 18,878), 1000 Genomes = 0.0048 (cohort size 2,504), and our population were Caucasian and predominantly born in the United Kingdom.

In the discovery cohort we assessed all individual bam files with the Integrated Genome Viewer to determine which rs140124801 alleles were present, blind of the fSNPd results. All individuals predicted to have the rs140124801 rare allele were Sanger sequenced and complete concordance was found. Primers were designed with Primer3 and are available on request. Genomic DNA of the 58 individuals in the replication cohort was Sanger sequenced to determine the SNP rs140124801 allele frequencies. The allele frequency and number of heterozygotes of rs140124801 was assessed in combined cohort of discovery and replication by Chi^2^ test with two tailed and Yates correction (for small numbers), using the more conservative control population allele frequency of 0.0072 for heterozygote carriers.

We assessed the effects on protein sequence and protein function of the *KCNG4* SNP rs140124801 alleles by use of bioinformatic resources within the Human Genome Browser, NCBI BLASTP for protein sequence comparisons and Conserved Domains (CD search) for detecting if the amino acid change occurred within a known protein domain and SIFT for pathogenicity prediction.

#### Modeling of KCNG4 alleles on the K_v_2.1/K_v_6.4 heterotetramer

We used the X-ray crystallography derived structure of rat K_V_2.1 homotetramer (RCSB Protein Data Bank ID: 3LNM) to model the effects of the rare allele of rs140124801 (Tao et al., 2010). Rat and human subunits form both K_V_2.1 homotetramers and 3:1 K_V_2.1:K_V_6.4 heterotetramers. Rat was the closest species to humans with a published K_V_2.1 protein structure. Rat and human K_V_2.1 proteins are 94% identical and 79% identical for K_V_6.4. However, restricting the alignment to the 78 amino-acid region physically adjacent to the K^+^ selectivity filter (the pore loop from transmembrane region 5 to transmembrane region 6, which includes the K^+^ selectivity filter) rat and human K_V_2.1, they are identical and rat and human K_V_6.4 is 96% identical (with no amino acid changes in the aliphatic pocket or selectivity filter).

We used structure 3LNM and the Cn3D software (Wang et al., 2000) to examine the K^+^ selectivity filter of the K_V_2.1 homotetramer to look at the sites of interaction of each of the four individual K_V_2.1 proteins, and produced images where proteins and individual amino acids were identifiable, or could be omitted from the whole tetrameric structure.

The K^+^ ion selectivity filter is formed by the peptide backbone carbonyl groups of the amino acids TVGYG of each of the four K_V_ subunits. This forms a narrow central channel through which potassium ions (K^+^) can flow out of the cell. Each K_V_ subunit forms an identical quarter of the tetramer structure about the selectivity filter region central pore. The K_V_2.1 homotetramer model reveals the side chains of the Valine and Tyrosine of each subunit selectivity filter TVGYG protruding into a highly conserved “aliphatic pocket” (with canonical sequence WWAIIS; see Figure 1C) in the adjacent K_V_ subunit. In this model the Valine-419 of K_V_6.4 can be accommodated identically compared with the equivalent Valine-374 of K_V_2.1. However, the larger aliphatic side chain of 419-Methionine in the K_V_6.4 SNP would not be able to be accommodated within the aliphatic pocket, and hence would disrupt the ion selectivity region of the K_V_2.1/2.1/2.1/6.4 heterotetramer.

#### DNA constructs and antibodies

A full-length human *KCNG4* cDNA clone was purchased from Source bioscience (IRCMp5012B0629D) and cloned in-house into a pcDNA3 based expression plasmid (CMV-genex-polioIRESmCherry) both with or without a C-terminal HA tag. The p.Val419Met mutation was introduced by site-directed mutagenesis (Stratagene) according to the manufacturer’s protocols and sequences of the plasmids were confirmed by Sanger Sequencing. The clone expressing K_V_2.1 alongside a nuclear GFP reporter in the pCAGGS-IRES2-nucEGFP vector has been described previously (Saitsu et al., 2015) and was a kind gift from Prof. Hiromoto Saitsu.

Antibodies used were anti-HA mouse monoclonal (12B12, #MMS-101P, Biolegend), anti-Na^+^/K^+^ ATPase rabbit monoclonal (ab76020, Abcam), anti-mCherry rat monoclonal (M11217), anti-β-actin mouse monoclonal (a2228, Sigma), anti-K_V_2.1 rabbit polyclonal (APC-012, Alomone), anti-K_V_6.4 mouse monoclonal (N458/10, NeuroMab), anti-K_V_2.1 mouse monoclonal (K89/34, ab192761, Abcam), and anti-HA rabbit monoclonal (C29F4 #3724, Cell Signaling).

#### Immunofluorescence analysis and confocal microscopy

HEK293 and HeLa cells were cultured on poly-L-lysine coated coverslips and transfected as described above. 48-hours after transfection, cells were fixed by 10 minutes incubation in 4% paraformaldehyde. Cells were permeabilized by 10 minutes incubation in 0.3% Triton X-100 solution followed by 30 minutes at room temperature in 5% BSA solution. Alternatively, when staining for Na^+^/K^+^ ATPase, cells were fixed and permeabilized by emersion in ice cold methanol. Fixed cells were then stained with primary antibodies for 1 hour in 5% BSA and fluorescent secondary antibody also for 1 hour. Secondary antibodies used were Alexa Fluor 488 donkey anti-mouse, Alexa Fluor 546 goat anti-rabbit, Alexa Fluor 546 donkey anti-mouse, Alexa Fluor 633 goat anti-mouse (all from Life Technologies). Coverslips were mounted onto glass slides using Prolong Diamond Antifade Mountant with DAPI (Molecular Probes). Cells were visualized with an LSM880 confocal microscope.

#### Co-immunoprecipitation

HEK293 cells were transfected with K_V_2.1 and K_V_6.4 plasmid constructs as described in the associated figures, and harvested after 3 days. Co-immunoprecipitation was carried out using the Dynabeads Co-Immuniprecipitation Kit (Life Technologies) according to the manufacturers protocols. Antibodies used were anti-HA mouse monoclonal (12B12, #MMS-101P, Biolegend), anti-K_V_2.1 rabbit polyclonal (APC-012, Alomone), anti K_V_2.1 mouse monoclonal (K89/34, ab192761, Abcam), and anti-HA rabbit monoclonal (C29F4 #3724, Cell Signaling). IP buffer supplied in the kit was supplemented with 80mM NaCl.

#### Electrophysiological characterization of KCNG4 SNP rs140124801 alleles and KCNB1 in HEK293 cells

Whole-cell recordings from transfected HEK293 cells were performed using 1-2.5 MΩ resistance fire-polished borosilicate glass electrodes filled with an internal pipette solution containing (in mM): KCl (110), K_4_-BAPTA (5), HEPES (10), MgCl_2_ (1), K_2_ATP (5), pH 7.3, 281 mOsm/kg. Cells were continuously superfused with bath solution containing (in mM): NaCl (145), KCl (4), HEPES (10), D-glucose (10), CaCl_2_ (1.8) MgCl_2_ (1), pH 7.4, 300 mOsm/kg, at room temperature (20-24°C). Potassium currents (*I*_K_) were recorded in voltage clamp mode using an Axopatch 200B connected through a Digidata 1440A A/D converter and pCLAMP software (version 10, Axon Instruments). The calculated linear leakage current was digitally subtracted offline for all current measurements. Potassium currents were elicited by step depolarisations from a holding potential of −90 mV to various test potentials. The voltage-dependence of activation was determined from tail currents recorded from a 200 ms voltage step to −60 mV following these various test potentials. The normalized tail currents were plotted against the voltage of the step depolarisations and fit with a Boltzmann function. A double pulse protocol was used to measure the voltage-dependence of steady-state inactivation. The protocol consisted of a 5 s prepulse that ranged between −110 to +40 mV from a holding potential of −90 mV followed by a 200 ms test pulse to +50 mV. Normalized currents during this test pulse were plotted against the prepulse voltage and fit with a Boltzmann function. The time course of recovery from inactivation was investigated by applying a 5 s prepulse to +20 mV from a holding potential of −90 mV and applying a 200 ms test pulse to +20 mV at various time intervals after the conditioning prepulse. Recoveries from inactivation time courses were fit to a single exponential function.

#### Single-cell qRT-PCR of mouse uterus innervating sensory neurons

Uterus innervating sensory neurons were retrograde labeled and the mRNA transcript expression for genes of interest determined using methodology previously described for other visceral organs (Hockley et al., 2019; Peiris et al., 2017; Prato et al., 2017). Female C57BL/6J mice (10-12 weeks) were used. Following laparotomy, 2 injections (~2.5 μl/injection) of Fast Blue (FB: 2% in saline) were made, one into each uterine horn adjacent to the cervix. Following recovery, animals were provided a soft, glucose-enriched diet and prophylactic post-operative analgesia (buprenorphine 0.05-0.1 mg kg^-1^). After 5-8 days, mice were killed by cervical dislocation and two primary cultures made from DRG T12-L2 (TL) and L5-S2 (LS), respectively. Dissected DRG were incubated in Lebovitz L-15 Glutamax (Thermo Fisher Scientific, UK) media containing 6 mg ml^-1^ bovine serum albumin (BSA, Sigma-Aldrich) and 1 mg ml^-1^ collagenase type 1A (Sigma-Aldrich, UK) for 15 min at 37°C in 5% CO_2_. After a further 30 min incubation in L-15 media containing 1mg ml^-1^ trypsin (Sigma-Aldrich) and 6 mg ml^-1^ BSA, ganglia were triturated and dissociated cell-containing supernatant collected by repeat brief centrifugation (5 × 500 *g*). TL and LS neurons were plated on poly-D-lysine coated coverslips (BD Biosciences, UK) and incubated in L-15 growth media (containing 2% penicillin/streptomycin, 24 mM NaHCO_3_, 38 mM glucose and 10% fetal bovine serum). Fluorescently labeled FB-positive cells were picked manually by pulled glass pipette into 9 μL mastermix (containing 5 μL CellsDirect 2 x reaction buffer (Invitrogen, UK), 2.5 μL 0.2 x primer-probe mix against genes of interest, 0.1 μL SUPERase-in (Ambion, USA), 1.2 μL TE buffer (Applichem, Germany) and 0.2 μL Superscript III Reverse Transcriptase-Platinum Taq mix (Invitrogen, UK)), bath samples were collected as negative controls and all samples immediately frozen on dry ice. Prior to 1:5 dilution in TE buffer, reverse transcription and preamplification of cDNA was performed by thermal cycling (50°C for 30 min, 95°C for 2 min, then 24 cycles of 95°C for 15 s, 60°C for 4 min). Gene-specific Taqman qPCR assays were then run (Taqman Assay ID: *Kcng4*, Mm01240890_m1; *Kcnb1*, Mm00492791_m1; *Trpv1*, Mm01246300_m1; *Scn10a*, Mm00501467_m1; *Gapdh*, Mm99999915_g1; Applied Biosystems) with the following thermal cycling protocol (50°C for 2 min, 95°C for 10 min, then 40 cycles of 95°C for 15 s, 60°C for 1 min). The expression of glyceraldehyde-3-phosphate dehydrogenase (*Gapdh*) acted as an internal positive control and was present in all single-cell RT-PCR products and absent in bath control samples. 15 uterine sensory neurons per region (TL and LS) per mouse (N = 3) were collected. In total, 90 neurons were collected, and photos taken for analysis of cell diameter. qPCR products were detected in 89 neurons and quantitative assessment of gene expression was determined by quantification cycle values less than the threshold of 35 considered positive.

#### Whole-cell patch-clamp recordings

Primary DRG cultures from C57BL/6J mice (8-10 weeks) were generated using the methodology described for single-cell qRT-PCR experiments with the following exceptions. From each mouse, DRG T10-S1 were dissected and pooled into a single primary culture. After trituration, in order to purify the DRG culture to improve transfection efficiency, dissociated cells were subjected to a 3.5% BSA (in L-15 media) density gradient centrifugation (20 mins at 20 *g*) and the supernatant discarded. The remaining purified dissociated neurons were washed once in L-15 growth media before resuspension in 100 μl of Mouse Neuron Nucleofector solution (Amaxa Mouse Neuron Nucleofector Kit, Lonza, UK) containing 4.5 μg plasmid of either wild-type K_V_6.4 or K_V_6.4-Met419 in a CMV-KCNG4-polioIRESmCherry expression cassette. Incorporation of the plasmid was achieved by electroporation (Program O-0005; Nucleofector IIb, Lonza, UK) and cells plated on poly-D-lysine/laminin coated coverslips (BD Biosciences, UK) and incubated at 37°C in 5% CO_2_ and L-15 growth media. Electrophysiology experiments were conducted 48-hours post-transfection, neurons positive for mCherry fluorescence were selected following excitation with a 572 nm LED (Cairn Research, UK).

To assess voltage-gated K^+^ currents, patch pipettes were pulled using a P-97 pipette puller (Sutter Instruments, USA) with typical resistances of 3-5 MΩ and back-filled with the pipette solution containing (in mM): KAspartate (110), KCl (30), MgCl_2_ (2), CaCl_2_ (1), Na_2_ATP (5), EGTA (2), cAMP (0.1), HEPES (10), pH 7.4. Recordings were obtained using a Multiclamp 700A amplifier (Molecular Devices, USA) in the voltage-clamp mode and digitised using a Digidata 1440A (Molecular Devices). Voltage errors were minimized using 70% series resistance compensation. Mouse neurons were continuously superfused with the bath solution containing (in mM): N-methyl-D-glucamine (NMDG; 140), KCl (5), MgCl_2_ (1), CaCl_2_ (1.8), glucose (10), HEPES (5), pH 7.4. The osmolality of both solutions was adjusted to 300-310 mOsm. Cells with series resistance values greater than 15 MΩ were omitted from analysis.

*I*_K_ activation and inactivation protocols (Figures 3 and S5) were applied after achieving whole-cell rupture. Using a rapid change perfusion system (Intracel EVH-9, UK), 100 nM Stromatoxin-1 (Alomone, Israel) in bath solution was applied to the cells for 3 minutes prior to repeating activation and inactivation protocols. Thus ScTx-sensitive *I*_K_ was determined by subtraction of the post-ScTx *I*_K_ from the pre-ScTx *I*_K_ in pClamp software (Molecular Devices). The voltage-dependence of *I*_K_ activation was fitted with the following Boltzmann equation: y = *t* / (1 + exp((V_50_ – E)/*k*)), where E is the applied voltage, V_50_ is the voltage at which 50% of the channels are activated, *t* is the top of the curve, and *k* is the slope factor. While the voltage-dependence of *I*_K_ inactivation was fitted with the sum of two Boltzmann equations: y = (*t*F / (1 + exp((_1_V_50_ - E) /_1_*k*)) + (t(1 - F) / (1 + exp((_2_V_50_-E)/_2_*k*), where E is the applied voltage, _1_V_50_ is the voltage at which 50% of the 1st component channels are inactivated, _2_V_50_ is the voltage at which 50% of the 2nd component channels are inactivated, *t* is the top of the curve, _1_*k* is the slope factor for the first component and _2_*k* for the second component, and F defines the relative component contribution.

For current clamp experiments a HEKA EPC-10 amplifier (Lambrecht, Germany) and the corresponding Patchmaster software were used. The extracellular solution contained (in mM): NaCl (140), KCl (4), MgCl_2_ (1), CaCl_2_ (2), glucose (4) and HEPES (10), pH 7.40. Patch pipettes, pulled as for *I*_K_ experiments, were filled with intracellular solution containing (in mM): KCl (110), NaCl (10), MgCl_2_ (1) EGTA (1), HEPES (10), Na_2_ATP (2), Na_2_GTP (0.5), pH 7.3. After gaining access to cells and entering current clamp mode the resting membrane potential of neurons was noted. Ramp depolarisation from 0 pA to 1 nA over a period of 1 s was first used to assess action potential threshold. A step protocol (Δ10 pA, 50 ms) was then used to confirm thresholds. The ability of neurons to fire multiple action potentials was assessed by applying a suprathreshold (2x the threshold determined by step protocol) for 500 ms. Lastly, capsaicin (1 μM in extracellular solution) sensitivity was assessed in voltage clamp mode; neurons that produced an inward current, time-locked to a 5 s application were considered responders. Only cells which fired action potentials and had a resting membrane potential less than or equal to −40 mV were taken through to analyses. Action potential parameters were measured from those evoked by the step protocol using Fitmaster software (HEKA) and IgorPro (Wavemetrics).

### Quantification and Statistical Analyses

For psychometric and quantitative sensory testing, statistical analyses were performed with R Studio (Version 1.1.442). The mean and standard deviation were generated for each outcome variable for test and control cohorts. Shapiro-Wilk tests and F-tests were used assess data normality and differences in variances. Differences between the means of each outcome variable in test and control cohorts were assessed using tests for two independent samples, using Student’s t test, Welch’s t test or Mann-Whitney U tests when the relevant assumptions were met. The level of statistical significance was adjusted using Sidak’s correction. The correction applied to multiple outcomes associated with each domain of assessments: questionnaires, CANTAB, sensory detection, pain thresholds and tolerance.

For statistical assessment of the genetic data collected in this study, enrichment of amino acid altering SNPs was assessed by exome sequencing, with exome v*cf.*, bam and bam.bai files iteratively analyzed to extract data on all SNPs in or near to exons, including the depth and quality of the sequence data, and the alleles detected (Stouffer et al., 2017). For each SNP the allele frequencies were compared to normal values derived from the 1000 genomes project and exome variant server, and deviations assessed for significance using a Chi-square test with two tails and Yates correction. The resulting *P value*s were subject separately to a Bonferroni and FDR correction, as approximately 100,000 SNPs were assessed in our fSNPd method.

We then focused only on ion channels, defined as being identified by the Gene Ontology Term GO:0005216 (423 genes, which were also hand curated and checked against a Pfizer/Neusentis database that had been shared with us). There were 28 SNPs found in ion channels and each was in a different gene; there was only one detected SNP in *KCNG4*. Eight of these SNPs were then eliminated because the protein change caused by the rare allele was common in the orthologous mammalian proteins. For the remaining 20 SNPs, we determined the allele frequency of each of the by use of the Integrated Genome Viewer examining the cohort’s exome bam files individually. This led to the elimination of 19 SNPs, 14 as the common allele frequency was 100% and program errors in assigning alleles within our discovery cohort had falsely appeared to show a deviation from 100%, and five because of misalignment of reads to homologous genes leading to errors in SNP allele calling and SNP allele frequency calculation.

Further statistical tests used to assess differences between K_V_6.4 and K_V_6.4-Met419 in the cellular and animal studies are unpaired t tests and ANOVA with Bonferroni’s multiple comparison post hoc test, as described in the relevant figure legends. Differences between groups were considered significant at a *P value* < 0.05, and were tested using GraphPad (Prism5.0, California, USA).

## Supplemental Information

Document S1. Figures S1–S6 and Tables S1, S2, S4, and S5

Table S3. SNP Allele Frequency Data, Related to Figure 1A List of all SNPs in discovery cohort that had a cohort allele frequency that deviated from the expected frequencies found in either the 1000 Genomes, European data or the Exome Variant server European data sets. For each SNP the following data are shown: its genomic location, number of cases and allele frequency for the rare allele in the research cohort and 1000 Genomes project and Exome Variant Server, p value with Bonferroni correction of deviation from expected, p value with false discovery rate correction of deviation from expected, gene in which the SNP change occurred (when occurring within a gene), effect of SNP rare allele base change and the position in c.DNA (when occurring in cDNA), SNP rare allele amino acid change and position (where occurring in a protein), the SNPs dbsnp nomenclature, the rare allele change Polyphen score and SIFT score.

Document S2. Article plus Supplemental Information

## References

[bib1] Bennett D.L., Clark A.J., Huang J., Waxman S.G., Dib-Hajj S.D. (2019). The Role of Voltage-Gated Sodium Channels in Pain Signaling. Physiol. Rev..

[bib2] Bergh I.H., Johansson A., Bratt A., Ekström A., Mårtensson L.B. (2015). Assessment and documentation of women’s labour pain: A cross-sectional study in Swedish delivery wards. Women Birth.

[bib3] Bertucci P., Arendt D. (2013). Somatic and visceral nervous systems - an ancient duality. BMC Biol..

[bib4] Bocksteins E. (2016). Kv5, Kv6, Kv8, and Kv9 subunits: No simple silent bystanders. J. Gen. Physiol..

[bib5] Bocksteins E., Snyders D.J. (2012). Electrically silent Kv subunits: their molecular and functional characteristics. Physiology (Bethesda).

[bib6] Bocksteins E., Raes A.L., Van de Vijver G., Bruyns T., Van Bogaert P.P., Snyders D.J. (2009). Kv2.1 and silent Kv subunits underlie the delayed rectifier K+ current in cultured small mouse DRG neurons. Am. J. Physiol. Cell Physiol..

[bib7] Bocksteins E., Labro A.J., Snyders D.J., Mohapatra D.P. (2012). The electrically silent Kv6.4 subunit confers hyperpolarized gating charge movement in Kv2.1/Kv6.4 heterotetrameric channels. PLoS ONE.

[bib8] Bocksteins E., Snyders D.J., Holmgren M. (2017). Independent movement of the voltage sensors in K_V_2.1/K_V_6.4 heterotetramers. Sci. Rep..

[bib9] Carvalho B., Zheng M., Aiono-Le Tagaloa L. (2013). Evaluation of experimental pain tests to predict labour pain and epidural analgesic consumption. Br. J. Anaesth..

[bib10] Carvalho B., Zheng M., Aiono-Le Tagaloa L. (2014). A prospective observational study evaluating the ability of prelabor psychological tests to predict labor pain, epidural analgesic consumption, and maternal satisfaction. Anesth. Analg..

[bib11] Chakrabarti S., Pattison L.A., Doleschall B., Rickman R.H., Blake H., Callejo G., Heppenstall P.A., St John Smith E. (2020). Intra-articular AAV-PHP.S mediated chemogenetic targeting of knee-innervating dorsal root ganglion neurons alleviates inflammatory pain in mice. Arthritis Rheumatol..

[bib12] Chung W.H., Hung S.I., Hong H.S., Hsih M.S., Yang L.C., Ho H.C., Wu J.Y., Chen Y.T. (2004). Medical genetics: a marker for Stevens-Johnson syndrome. Nature.

[bib13] Cuello L.G., Jogini V., Cortes D.M., Perozo E. (2010). Structural mechanism of C-type inactivation in K(+) channels. Nature.

[bib14] Escoubas P., Diochot S., Célérier M.L., Nakajima T., Lazdunski M. (2002). Novel tarantula toxins for subtypes of voltage-dependent potassium channels in the Kv2 and Kv4 subfamilies. Mol. Pharmacol..

[bib15] Gruss L.T., Schmitt D. (2015). The evolution of the human pelvis: changing adaptations to bipedalism, obstetrics and thermoregulation. Philos. Trans. R. Soc. Lond. B Biol. Sci..

[bib16] Haestier A., Hamilton S., Chilvers R.J. (2012). Labour outcomes in siblings with channelopathy associated insensitivity to pain due to bi-alleleic SCN9A mutations. Obstet. Med..

[bib17] Herweijer G., Kyloh M., Beckett E.A., Dodds K.N., Spencer N.J. (2014). Characterization of primary afferent spinal innervation of mouse uterus. Front. Neurosci..

[bib18] Hockley J.R.F., Taylor T.S., Callejo G., Wilbrey A.L., Gutteridge A., Bach K., Winchester W.J., Bulmer D.C., McMurray G., Smith E.S.J. (2019). Single-cell RNAseq reveals seven classes of colonic sensory neuron. Gut.

[bib19] Jones L., Othman M., Dowswell T., Alfirevic Z., Gates S., Newburn M., Jordan S., Lavender T., Neilson J.P. (2012). Pain management for women in labour: an overview of systematic reviews. Cochrane Database Syst. Rev..

[bib20] Ju H., Jones M., Mishra G. (2014). The prevalence and risk factors of dysmenorrhea. Epidemiol. Rev..

[bib21] Labor S., Maguire S. (2008). The Pain of Labour. Rev. Pain.

[bib22] Levett K.M., Smith C.A., Bensoussan A., Dahlen H.G. (2016). Complementary therapies for labour and birth study: a randomised controlled trial of antenatal integrative medicine for pain management in labour. BMJ Open.

[bib23] Loeser J.D., Treede R.D. (2008). The Kyoto protocol of IASP Basic Pain Terminology. Pain.

[bib24] Malin S., Molliver D., Christianson J.A., Schwartz E.S., Cornuet P., Albers K.M., Davis B.M. (2011). TRPV1 and TRPA1 function and modulation are target tissue dependent. J. Neurosci..

[bib25] Maul A. (2007). An evolutionary interpretation of the significance of physical pain experienced by human females: defloration and childbirth pains. Med. Hypotheses.

[bib26] Melzack R. (1984). The myth of painless childbirth (the John J. Bonica lecture). Pain.

[bib27] Melzack R. (1987). The short-form McGill Pain Questionnaire. Pain.

[bib28] Melzack R., Schaffelberg D. (1987). Low-back pain during labor. Am. J. Obstet. Gynecol..

[bib29] Mis M.A., Yang Y., Tanaka B.S., Gomis-Perez C., Liu S., Dib-Hajj F., Adi T., Garcia-Milian R., Schulman B.R., Dib-Hajj S.D., Waxman S.G. (2019). Resilience to Pain: A Peripheral Component Identified Using Induced Pluripotent Stem Cells and Dynamic Clamp. J. Neurosci..

[bib30] Mitchell L.A., MacDonald R.A., Brodie E.E. (2004). Temperature and the cold pressor test. J. Pain.

[bib31] Nahorski M.S., Maddirevula S., Ishimura R., Alsahli S., Brady A.F., Begemann A., Mizushima T., Guzmán-Vega F.J., Obata M., Ichimura Y. (2018). Biallelic UFM1 and UFC1 mutations expand the essential role of ufmylation in brain development. Brain.

[bib32] National Perinatal Epidemiology Unit (2014). Safely delivered: a national survey of women’s experience of maternity care. https://www.npeu.ox.ac.uk/.

[bib33] Ottschytsch N., Raes A.L., Timmermans J.P., Snyders D.J. (2005). Domain analysis of Kv6.3, an electrically silent channel. J. Physiol..

[bib34] Peiris M., Hockley J.R., Reed D.E., Smith E.S.J., Bulmer D.C., Blackshaw L.A. (2017). Peripheral K_V_7 channels regulate visceral sensory function in mouse and human colon. Mol. Pain.

[bib35] Prato V., Taberner F.J., Hockley J.R.F., Callejo G., Arcourt A., Tazir B., Hammer L., Schad P., Heppenstall P.A., Smith E.S., Lechner S.G. (2017). Functional and Molecular Characterization of Mechanoinsensitive “Silent” Nociceptors. Cell Rep..

[bib36] Prezant T.R., Agapian J.V., Bohlman M.C., Bu X., Oztas S., Qiu W.Q., Arnos K.S., Cortopassi G.A., Jaber L., Rotter J.I. (1993). Mitochondrial ribosomal RNA mutation associated with both antibiotic-induced and non-syndromic deafness. Nat. Genet..

[bib37] Robbins T.W., James M., Owen A.M., Sahakian B.J., Lawrence A.D., McInnes L., Rabbitt P.M. (1998). A study of performance on tests from the CANTAB battery sensitive to frontal lobe dysfunction in a large sample of normal volunteers: implications for theories of executive functioning and cognitive aging. Cambridge Neuropsychological Test Automated Battery. J. Int. Neuropsychol. Soc..

[bib38] Rolke R., Baron R., Maier C., Tölle T.R., Treede R.D., Beyer A., Binder A., Birbaumer N., Birklein F., Bötefür I.C. (2006). Quantitative sensory testing in the German Research Network on Neuropathic Pain (DFNS): standardized protocol and reference values. Pain.

[bib39] Rolke R., Magerl W., Campbell K.A., Schalber C., Caspari S., Birklein F., Treede R.D. (2006). Quantitative sensory testing: a comprehensive protocol for clinical trials. Eur. J. Pain.

[bib40] Saitsu H., Akita T., Tohyama J., Goldberg-Stern H., Kobayashi Y., Cohen R., Kato M., Ohba C., Miyatake S., Tsurusaki Y. (2015). De novo KCNB1 mutations in infantile epilepsy inhibit repetitive neuronal firing. Sci. Rep..

[bib41] Scheier M.F., Carver C.S., Bridges M.W. (1994). Distinguishing optimism from neuroticism (and trait anxiety, self-mastery, and self-esteem): a reevaluation of the Life Orientation Test. J. Pers. Soc. Psychol..

[bib42] Sherwood C.C., Bauernfeind A.L., Bianchi S., Raghanti M.A., Hof P.R. (2012). Human brain evolution writ large and small. Prog. Brain Res..

[bib43] Stevens E.B., Stephens G.J. (2018). Recent advances in targeting ion channels to treat chronic pain. Br. J. Pharmacol..

[bib44] Stevens N.R., Hamilton N.A., Wallston K.A. (2011). Validation of the multidimensional health locus of control scales for labor and delivery. Res. Nurs. Health.

[bib45] Stouffer K., Nahorski M., Moreno P., Sarveswaran N., Menon D., Lee M., Geoffrey Woods C. (2017). Functional SNP allele discovery (fSNPd): an approach to find highly penetrant, environmental-triggered genotypes underlying complex human phenotypes. BMC Genomics.

[bib46] Sullivan M.J.L., Bishop S.R., Pivik J. (1995). The Pain Catastrophizing Scale: Development and validation. Psychol. Assess..

[bib47] Tao X., Lee A., Limapichat W., Dougherty D.A., MacKinnon R. (2010). A gating charge transfer center in voltage sensors. Science.

[bib48] Tsantoulas C., Zhu L., Yip P., Grist J., Michael G.J., McMahon S.B. (2014). Kv2 dysfunction after peripheral axotomy enhances sensory neuron responsiveness to sustained input. Exp. Neurol..

[bib49] Tsantoulas C., Denk F., Signore M., Nassar M.A., Futai K., McMahon S.B. (2018). Mice lacking Kcns1 in peripheral neurons show increased basal and neuropathic pain sensitivity. Pain.

[bib50] Vargas A., Vargas A., Hernández-Paz R., Sánchez-Huerta J.M., Romero-Ramírez R., Amezcua-Guerra L., Kooh M., Nava A., Pineda C., Rodríguez-Leal G., Martínez-Lavín M. (2006). Sphygmomanometry-evoked allodynia--a simple bedside test indicative of fibromyalgia: a multicenter developmental study. J. Clin. Rheumatol..

[bib51] Wang Y., Geer L.Y., Chappey C., Kans J.A., Bryant S.H. (2000). Cn3D: sequence and structure views for Entrez. Trends Biochem. Sci..

[bib52] Wheeler D.W., Lee M.C.H., Harrison E.K., Menon D.K., Woods C.G. (2014). Case Report: Neuropathic pain in a patient with congenital insensitivity to pain. F1000Res..

[bib53] Whitburn L.Y., Jones L.E., Davey M.A., Small R. (2017). The meaning of labour pain: how the social environment and other contextual factors shape women’s experiences. BMC Pregnancy Childbirth.

[bib54] Zeisel A., Hochgerner H., Lonnerberg P., Johnsson A., Memic F., van der Zwan J., Haring M., Braun E., Borm L.E., La Manno G. (2018). Molecular Architecture of the Mouse Nervous System. Cell.

[bib55] Zhong X.Z., Abd-Elrahman K.S., Liao C.H., El-Yazbi A.F., Walsh E.J., Walsh M.P., Cole W.C. (2010). Stromatoxin-sensitive, heteromultimeric Kv2.1/Kv9.3 channels contribute to myogenic control of cerebral arterial diameter. J. Physiol..

[bib56] Zigmond A.S., Snaith R.P. (1983). The hospital anxiety and depression scale. Acta Psychiatr. Scand..

