# Supplementary material for: Human Labor Pain Is Influenced by the Voltage-Gated Potassium Channel KV6.4 Subunit
Source: Cell Rep. 2020 Jul 21;32(3):107941. doi: 10.1016/j.celrep.2020.107941 (PMC7383234; doi:10.1016/j.celrep.2020.107941)
Supplement: Document S1. Figures S1–S6 and Tables S1, S2, S4, and S5 [file mmc1.pdf]

Cell Reports, Volume *32*

Supplemental Information

Human Labor Pain Is Influenced by the Voltage-Gated Potassium Channel K_V6.4 Subunit

Michael C. Lee, Michael S. Nahorski, James R.F. Hockley, Van B. Lu, Gillian Ison, Luke A. Pattison, Gerard Callejo, Kaitlin Stouffer, Emily Fletcher, Christopher Brown, Ichrak Drissi, Daniel Wheeler, Patrik Ernfors, David Menon, Frank Reimann, Ewan St. John Smith, and C. Geoffrey Woods

Study A DNA Sampling

Inclusion Criteria

Females who are

- Aged 18 years and above
- Able to communicate in English
- Caucasian
- Able to provide written and informed consent
- Who experienced term (beyond 37 week gestation) spontaneous vaginal delivery as nulliparous partituents
- Were healthy during the gestation of the first born

Exclusion Criteria

Females who

- Requested **or** was provided systemic or regional analgesia, including inhalation anaesthetics, spinal or epidurals and opioids (any routes) during delivery of their first child
- Reported having no opportunity for labour analgesia for any reason.
- Required assisted vaginal delivery, including use of Ventouse or forceps for their first child
- Had diabetes or hypertension induced by pregnancy of their first born
- Have known neurological (including channelopathies causing congenital insensitivity to pain) or psychiatric impairments

Study B Psychometrics, sensory, pain threshold and tolerance assesments

Inclusion criteria

Females

- who donated DNA in Study A or their corresponding controls

Exclusion criteria

Females who

- are pregnant or breast-feeding
- have any rash, broken skin or skin irregularities where sensory testing is performed
- any underlying medical condition or taking any drug that in the opinion of the investigator will interfere with quantitative sensory testing

Table S1. Eligibility criteria for Study A and Study B. *Related to: STARS Methods, 'Human case ascertainment and recruitment'*

Variable	Test cohort n	Test cohort mean	Test cohort SD	Control cohort n	Control cohort mean	Control cohort SD	*P*	CI5	CI95
**Questionnaires**									
HADS (Anxiety)	39	6.05	2.33	33	6.88	3.57	0.25845	-0.62534	2.28035
HADS (Depression)	39	2.10	1.37	33	2.48	2.18	0.77621	-0.99998	0.99996
PCS (Total)	39	9.56	6.97	33	11.18	7.50	0.41189	-1.99996	5.00002
MHLC (Internal)	39	26.59	3.23	33	26.85	3.23	0.73576	-1.26431	1.78179
MHLC (Chance)	39	17.31	5.40	33	18.48	3.92	0.15776	-0.99993	4.00008
MHLC (Powerful Others)	39	14.44	4.06	33	14.97	4.65	0.60498	-1.51513	2.58273
LOTR (Total)	39	17.46	4.60	33	16.97	4.61	0.65036	-2.99994	1.99996
**Computerized cognitive assessments (CANTAB)**									
**Motor Screening Task**									
Mean latency	38#	761.3079	447.9984	30#	687.06	135.79	0.85586	-64.69994	43.80002
Mean error	38#	7.208883	1.562239	30#	7.02	1.84	0.42846	-1.16448	0.50038
**Rapid Visual Information Processing (RVP)**									
RVP A'	36#	0.930904	0.056894	30#	0.92	0.04	0.16740	-0.04706	0.00919
RVP B'	35#	0.890124	0.333482	29#	0.95	0.05	0.47025	-0.01785	0.03418
**Spatial Working Memory**									
Strategy	38#	27.97	8.19	30#	30.10	6.01	0.29844	-1.00001	5.00003
Total errors	38#	16.39	15.99	30#	19.67	15.51	0.22973	-2.00006	10.99998
**Intra-Extra Dimensional Set Shift**									
Total errors (adjusted)	37#	18.73	16.45	30#	19.37	16.92	0.45651	-2.00000	3.99998
Stages completed	37#	8.81	0.57	30#	8.70	0.70	0.41987	-0.00003	0.00004
Total trials (adjusted)	37#	83.89	29.30	30#	84.77	29.12	0.51143	-3.99997	6.99994
**One Touch Stockings of Cambridge**									
Mean choices to correct	37#	1.09	0.07	30#	1.20	0.23	0.06084	-0.00005	0.10006
Mean latency to correct	37#	9689.43	4320.31	30#	10839.52	3998.01	0.06460	-70.24996	3115.09998

Table S2. Psychometric results for Study B. *Related to: STARS Methods, 'Clinical questionnaires, cognitive and sensory testing & Cambridge Neuropsychological Test Automated Battery (CANTAB)*

HADS, Hospital Anxiety and Depression Scale; PCS, Pain Catastrophising Scale; MHLC, Multi-dimensional Health Locus of Control; Life Orientation Test-Revised (LOTR). n, number of participants; #equipment unavailable/failure; SD, standard deviation; CI5-CI95, 5-95% confidence interval.

Table S3 [Provided as '*Table S3 SNP allele frequency data.xlsx*']

List of all SNPs in discovery cohort that had a cohort allele frequency that deviated from the expected frequencies found in either the 1000 Genomes, European data or the Exome Variant server European data sets. *Related to: Figure 1A*.

For each SNP the following data is shown; its genomic location, number of cases and allele frequency for the rare allele in the research cohort and 1000 Genomes project and Exome Variant Server, p value with Bonferroni correction of deviation from expected, p value with false discovery rate correction of deviation from expected, gene in which the SNP change occurred (when occurring within a gene), effect of SNP rare allele base change and the position in c.DNA (when occurring in cDNA), SNP rare allele amino acid change and position (where occurring in a protein), the SNPs dbsnp nomenclature, the rare allele change Polyphen score and SIFT score.

**A** **Pain threshold**	**KCNG4+** n	mean	SD	**KCNG4 -** n	mean	SD	P unadjusted	*P* adjusted*	CI5	CI95
Heat (°C)	3	10.1	5.00	69	14.2	9.10	0.31000	NA	-17.3	9.0
Cold (°C)	3	43.8	3.00	69	43.3	3.20	0.80000	NA	-7.2	8.2
Cuff-pressure (mmHg)	3	196.2	13.80	69	139.7	56.10	0.00290	0.0090	29.7	83.4

**B** **Pain threshold**	**Test cohort (KCNG4+ individuals excluded)** n	mean	SD	**Control cohort** n	mean	SD	P unadjusted	*P* adjusted*	CI5	CI95
Cuff-pressure (mmHg)	3	164.2	56.20	33	113.0	9.30	0.00008	0.0005	27.2	75.1

Table S4. *Related to: Table 1*

(A) Effect of the rare allele of *KCNG4* on pain thresholds. *KCNG4+*, individuals who possess the rare allele, *KCNG4-*, controls who do not possess the rare allele; n, number of participants; SD, standard deviation; * Sidak's correction; CI5-CI95, 5-95% confidence interval. **(B)** Comparison of the Test cohort (women who do not possess the rare *KCNG4* allele and did not require analgesic during nulliparous labour) and Control cohort. n, number of participants; SD, standard deviation; * Sidak's correction; CI5-CI95, 5-95% confidence interval.

A	K_V6.4	K_V6.4-Met419
n	8	7
Capacitance (pF)	22.9 ± 1.4	23.2 ± 1.8
Access resistance (MΩ)	8.2 ± 1.1	7.8 ± 0.9
Activation		
$V_{1/2}$ (mV)	-5.4 ± 1.8	-9.8 ± 1.1
k	8.6 ± 1.5	8.9 ± 0.9
Inactivation		
1st component		
$V_{1/2}$ (mV)	-0.8 ± 29.5	-36.2 ± 3.3
k	-46.1 ± 25.6	-63.9 ± 26.5
2nd component		
$V_{1/2}$ (mV)	-60.2 ± 6.6	-33.8 ± 2.1**
k	-29.6 ± 8.1	-26.4 ± 15.6

B	K_V6.4-Met419	K_V6.4	Untransfected
n	10	8	6
RMP (mV)	-50.10 ± 2.05	-47.33 ± 1.14	-46.00 ± 2.14
Capacitance (pF)	41.54 ± 10.17	31.53 ± 3.69	21.55 ± 4.78
Ramp Threshold (pA)	248.60 ± 50.33*	91.56 ± 16.74	112.50 ± 32.51
Number of ramp AP	12.70 ± 3.48	10.22 ± 2.47	15.50 ± 4.79
Step Threshold (pA)	172.00 ± 34.44*	61.11 ± 12.18	88.33 ± 13.76
Amplitude (mV)	75.20 ± 5.20	76.66 ± 6.72	59.87 ± 4.62
HPD (ms)	3.79 ± 0.81	5.69 ± 1.26	3.71 ± 0.62
AHP Duration (ms)	16.48 ± 1.65	31.53 ± 7.35	17.17 ± 3.16
AHP_{50} (ms)	8.52 ± 0.75	10.32 ± 2.15	8.75 ± 1.40
AHP Amplitude (mV)	18.49 ± 1.80	15.92 ± 1.97	17.95 ± 2.90
AP Freq @ 2xThr	6.40 ± 1.17	3.00 ± 0.73	4.33 ± 2.44

Table S5. *Related to: STARS Methods, 'Whole-cell patch-clamp recordings'*

(A) Electrophysiological characteristics of mouse sensory neurons transfected with wild-type Kv6.4 and Kv6.4-Met419. ** $P < 0.01$, mean ± SEM **(B)** Action potential parameters of mouse sensory neurones transfected with wild-type KV6.4 or Kv6.4-Met419 and untransfected cells from current clamp experiments. RMP, resting membrane potential, AP, action potential, HPD, half peak duration, AHP, afterhyperpolarisation duration, Thr,, threshold, Freq., frequency. *P <0.05, mean ± SEM

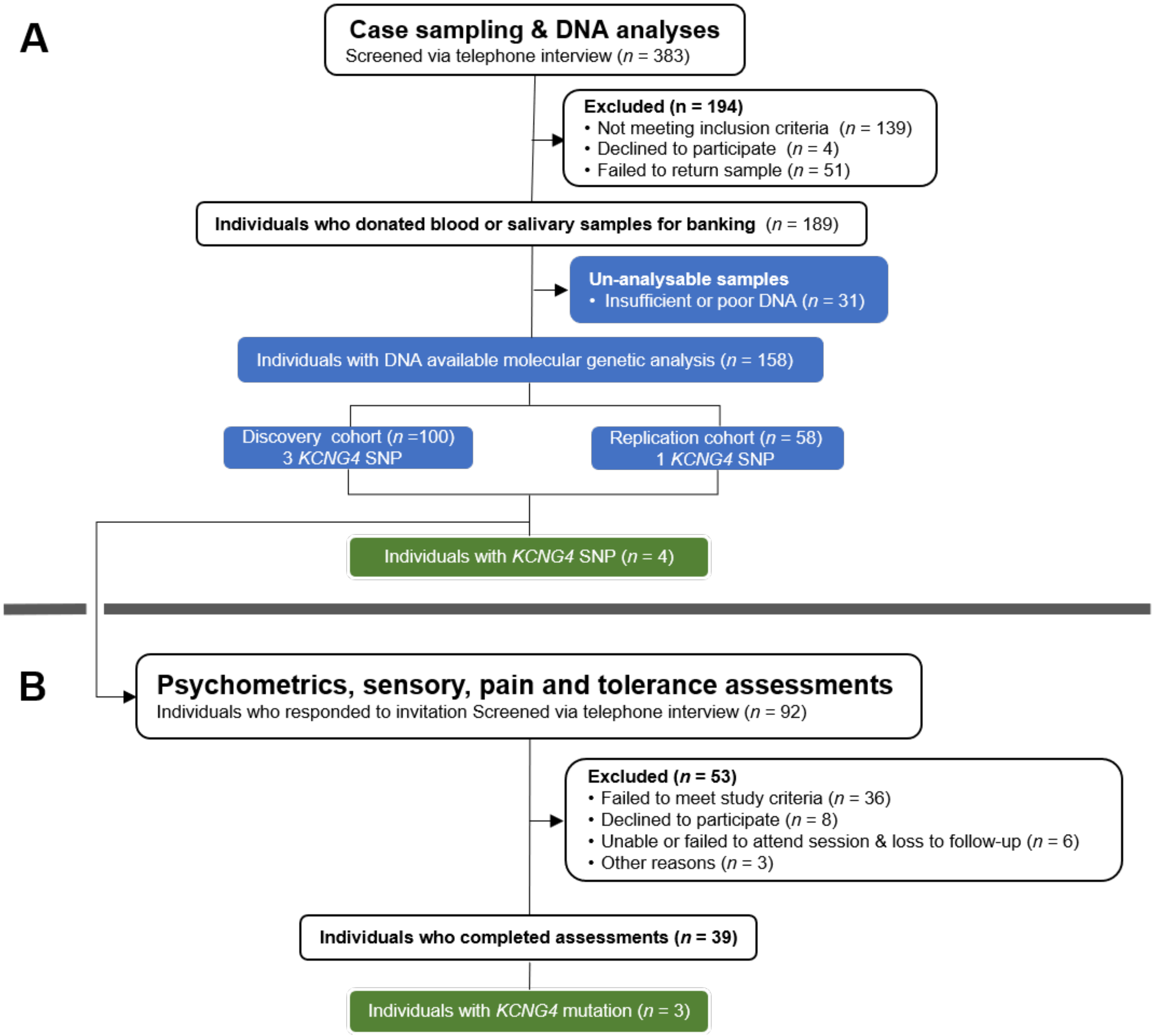

Figure S1 Flow-chart illustrating the recruitment and screening of participants. *Related to STAR Methods, 'Human case ascertainment and recruitment'*

(A) genetic sampling and **(B)** the subset of those participants who underwent psychometric, sensory and pain (threshold and tolerance) assessments. Blue rectangles indicate handling, processing and analyses of DNA samples that were donated by participants. Green rectangles indicated number of individuals assessed or DNA analysed with *KCNG4* mutation. n, number of samples or individuals.

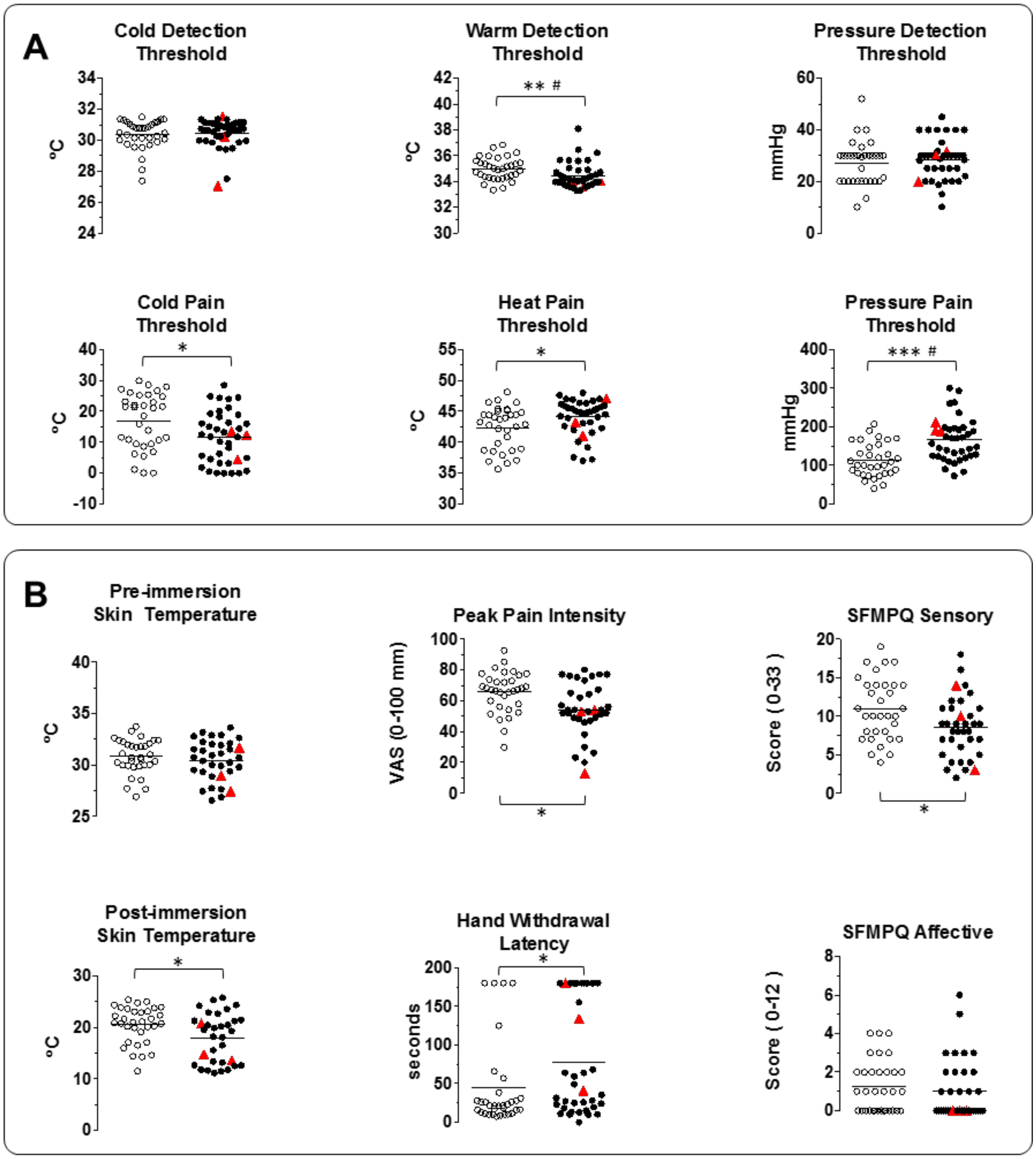

Figure S2 Sensory detection, pain threshold and tolerance assessments. *Related to: Table 1*

(A) Thresholds for sensory detection and pain for heat, cold and cuff pressure. (B) Testing of pain tolerance to hand immersion in cold water. Left-sided graphs: skin temperatures pre- and post-immersion. Middle graphs: withdrawal latency and ratings of peak pain experienced during hand immersion. Bottom graphs: ratings of the sensory and affective qualities of pain experienced with the SFMPQ. Clear circles indicate individuals in control cohort, and filled circles indicate those in the test cohort. The three individuals with KV6.4 p.Val419Met are indicated by red triangles. Horizontal lines represent the mean for each cohort. * P<0.05, ** P<0.01 *** P<0.001; # Sidak adjusted P <0.05

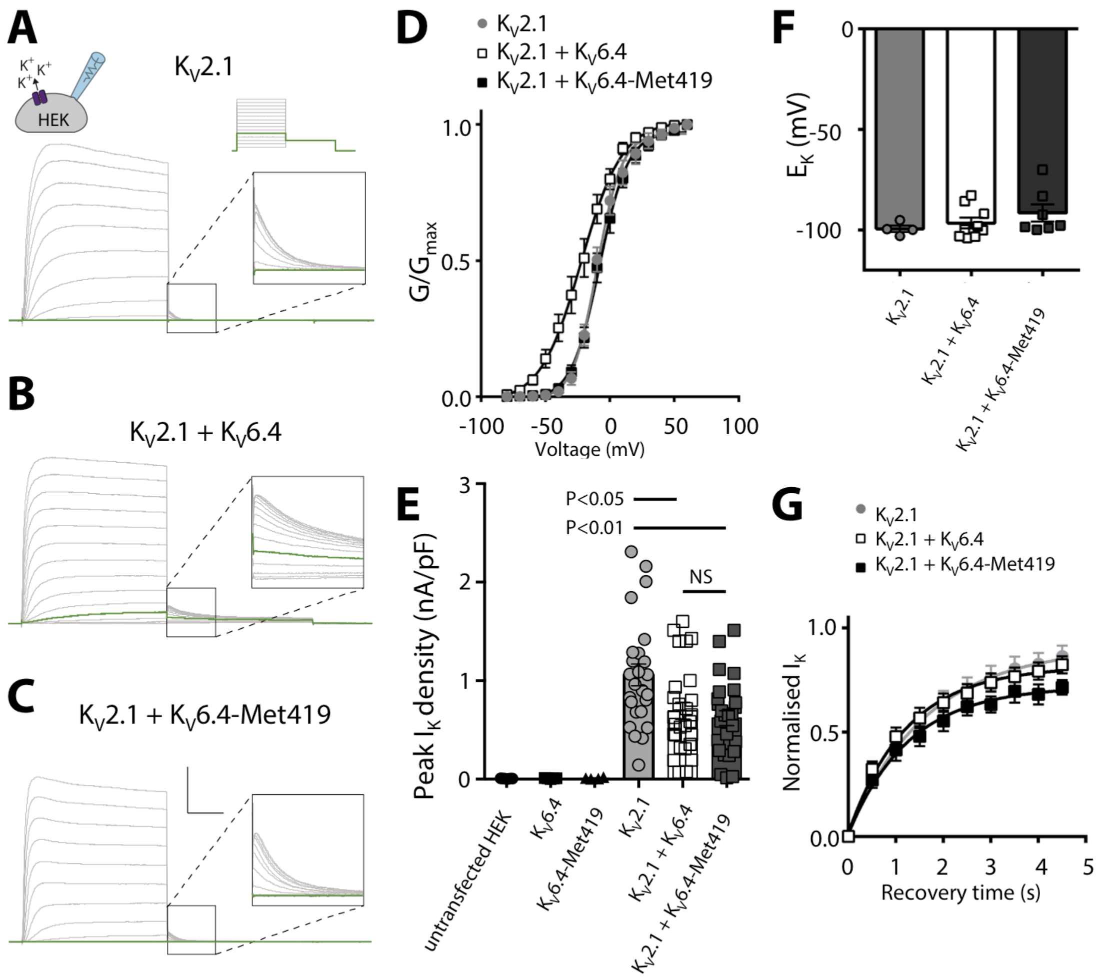

Figure S3 Supporting electrophysiology data for the functional effects of Kv6.4 and Kv6.4-Met419 on Kv2.1 currents in HEK293 cells. Representative current recordings to determine Kv2.1. *Related to: Fig. 1*

(A), Kv2.1/Kv6.4 (B), Kv2.1/Kv6.4-Met419 (C) channel activation properties. The applied voltage protocols are illustrated above the currents shown in (A). Vertical scale bar is 10 nA, horizontal scale bar is 50 ms. Green traces indicate currents recorded during the −40 mV prepulse. D. Voltage-dependence of activation of Kv2.1 (grey filled circles, n = 13), Kv2.1/Kv6.4 (open squares, n = 14), and Kv2.1/Kv6.4-Met419 (black squares, n = 13). The voltage-dependence of activation was determined by normalising tail currents at −60 mV as a function of a prepulse from −80 to +60 mV, in +10 mV increments. Solid lines represent the Boltzmann fitted curves. (E) Peak K^+ current density obtained from +30 mV step of voltage protocol. Bars indicate mean values, error bars indicate SEM. First three groups, n = 4-7 from 2 independent experiments, last three groups, n = 25-27 from 5 independent experiments. (F) Reversal potential obtained from a linear fit of tail currents from −10 mV to a series of voltage steps from −140 to −50 mV, in +10 mV increments. Bars indicate mean values, error bars indicate SEM, n = 4-9. (G) Recovery time from inactivation of Kv2.1 (grey filled circles, n = 6), Kv2.1/Kv6.4 (open squares, n = 10), and Kv2.1/Kv6.4-Met419 (black squares, n = 9). Relative peak current plotted from a 200 ms test pulse to +20 mV at various time intervals following a 5 s prepulse to +20 mV. Solid lines represent exponential fitted curves. Error bars represent SEM

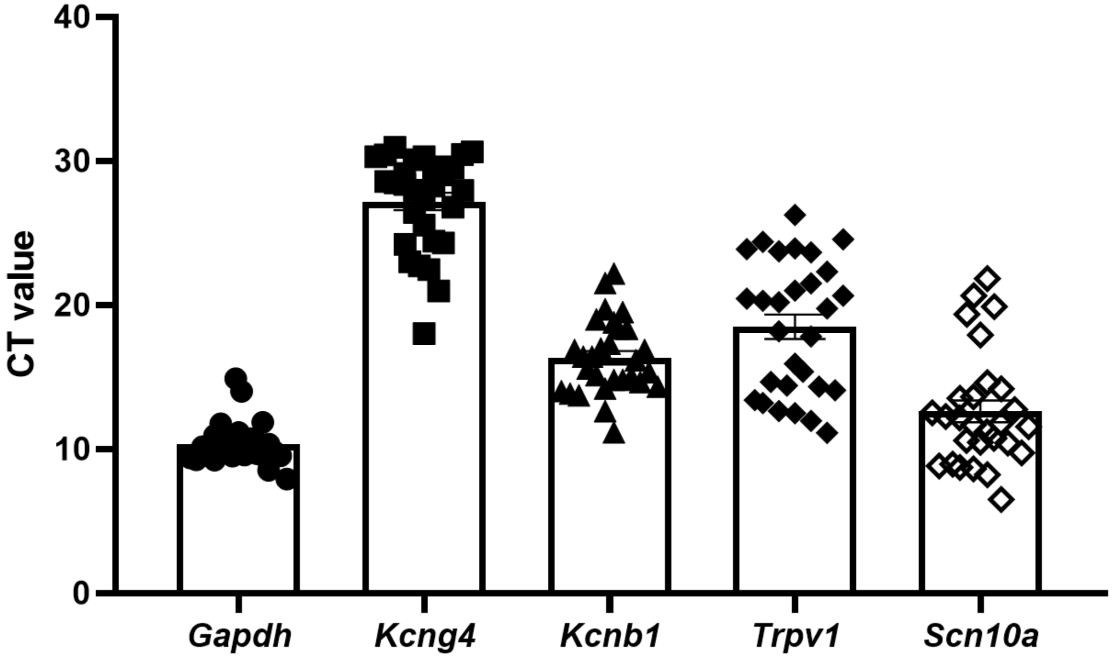

Figure S4 Mean raw cycle threshold (CT) values of *Kcng4*-positive mouse uterus-innervating sensory neurons. *Related to STAR Methods, 'Single-cell qRT-PCR of mouse uterus innervating sensory neurons'*

Data are shown (n=30 cells) for each gene assessed by single cell quantitative PCR analysis. Cells with CT values for specific genes above the quantification threshold of 35 were considered negative and not graphed. Error bars represent SEM.

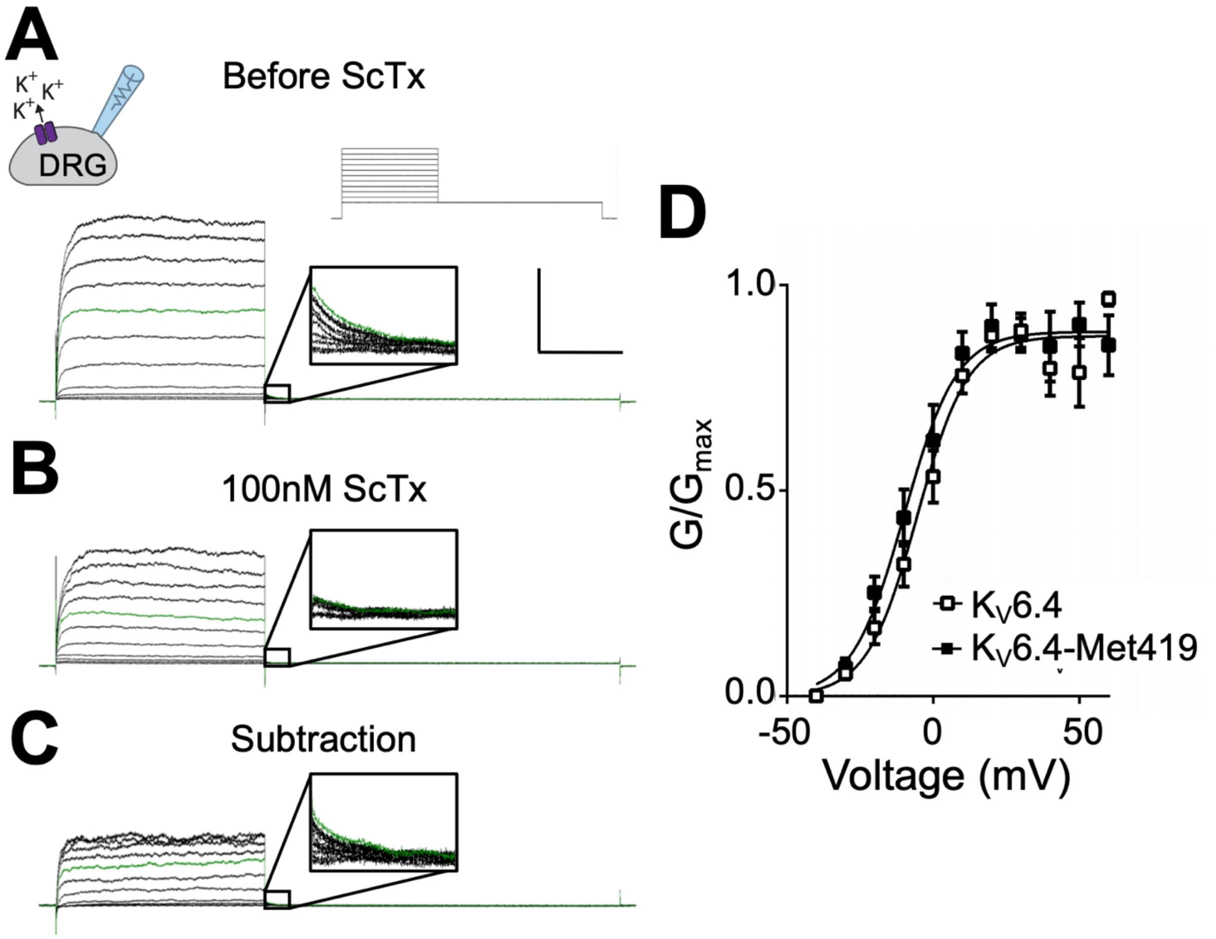

Figure S5 Effect of Kv6.4 and Kv6.4-Met419 on the voltage dependence of activation of the stromatoxin-1-sensitive current in mouse sensory neurons. *Related to STAR Methods: 'Whole-cell patch-clamp recordings'*

(A) Representative I_K recordings produced by *inset* voltage protocol in the absence and presence of 100nM ScTx (B). (C) The ScTx-sensitive I_K is isolated by subtraction of B from A. Expanded tail currents are shown for all three representative traces, each *inset* is 50 ms by 450 pA. The green tracing in A, B and C represent the current at +20 mV. (D) Activation curve of the ScTx-sensitive I_K obtained from mouse sensory neurons transfected with either wild-type Kv6.4 (n = 8) or Kv6.4-Met419 (n = 7). In both cases a Boltzmann function was fit to the data. Error bars represent SEM

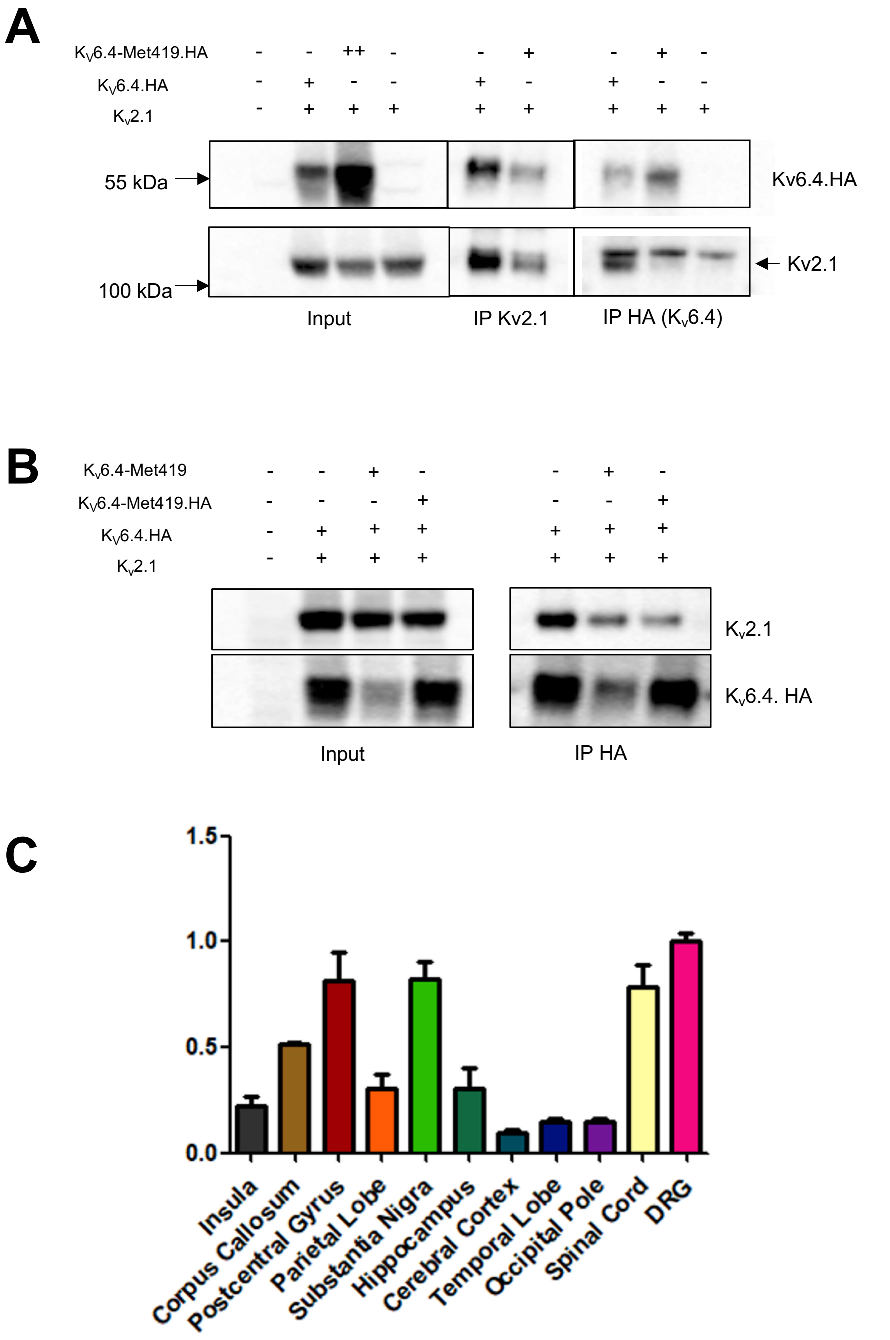

Figure S6. Data supporting lack of heterotetramerisation of Kv6.4-Met419 with Kv2.1 *Related to STAR Methods: 'Co-immunoprecipitation'*

(A) Co-immunoprecipitation experiments showing absence of Kv2.1 binding to Kv6.4-Met419 is not due to Kv6.4-Met419 lack of stability. There is significantly reduced binding of Kv6.4-Met419 to Kv2.1 even when significantly overexpressed compared to Kv6.4. This blot also confirms that HA antibody does not pull down Kv2.1 in the absence of Kv6.4 expression. (B) Co-immunoprecipitation experiment for Kv6.4 and Kv2.1 demonstrating that there is similar reduced binding for Kv6.4 to Kv2.1 in the heterozygous mutant state, whether or not the Kv6.4-Met419 is tagged with HA or not. (C) Expression levels of *KCNG4* in different human brain regions, the spinal cord and DRG (dorsal root ganglion). The graph displays the mean of three mRNA/cDNA conversions, assessed by TaqMan qPCR normalised to a *GAPDH* control and compared with the highest expressing tissue, the dorsal root ganglion (DRG). Error bars represent SEM.
